# Emerging roles of microRNAs and other non-coding transcriptome in muscular dystrophies

**DOI:** 10.1186/s41232-026-00415-7

**Published:** 2026-04-11

**Authors:** Farah Gamal Abdelrehim, Zade Sadek, Salma A. Fahim, Nada El-Ekiaby, Ahmed Ihab Abdelaziz, Injie Omar Fawzy

**Affiliations:** 1 School of Medicine, Newgiza University, Giza, Egypt; 2 Biotechnology Program, The American University in Cairo, Cairo, Egypt

**Keywords:** Non-coding transcriptome, Muscle dystrophy, Regeneration, Post-translational modifications, NcRNA, Neuromuscular diseases, Myogenesis, Degeneration, Inflammation

## Abstract

Muscular dystrophies (MDs) are a set of neuromuscular diseases characterized by progressive muscle weakness and wasting. Their pathophysiology entails several aberrant genetic pathways including the perturbation of microRNA (miRNA) and other non-coding RNA (ncRNA) levels and functions, and the subsequent dysregulation of their downstream targets. In healthy tissue, ncRNAs exert their influence by fine-tuning physiological mechanisms. However, in dystrophic conditions, these ncRNAs become involved in modulation of pathological mechanisms. The main pathomechanism themes that involve ncRNAs and proteins in MD are myogenesis insufficiency, structural instability, destructive pathways, and signaling failure. This review attempts to delineate all the major contributory ncRNAs, particularly miRNAs, as well as their associated proteins involved in disease initiation, maintenance, and outcomes across the spectrum of MD subtypes.

## Introduction

Muscular dystrophies (MDs) are a set of inherited neuromuscular disorders characterized by progressive muscle weakness and wasting [1]. There are over 30 different subtypes of MD and they vary widely in onset, etiology, presentation, distribution, and prognosis [2]. Their main drivers are genetic mutations which lead to various pathological mechanisms such as disturbed muscle cell integrity, faulty homeostatic mechanisms, defective cellular repair processes, aberrant signaling, metabolic inadequacies, and many others [3].

In recent decades, an emergent entity has garnered widespread attention due to its ubiquitous involvement and prevailing influence over all aspects of MD pathophysiology. This entity is the ever-expanding set of non-coding RNAs (ncRNAs). What was once referred to as “junk DNA” is now acclaimed as one of the most important driving forces in almost all disease [4]. The term ncRNA refers to all RNA that does not encode a protein, but rather, are alternatively spliced and/or processed in more nuanced ways [5]. There are various forms of ncRNA including microRNA (miRNA), small interfering RNA (siRNA), circular RNA (circRNA), long non-coding RNA (lncRNA), and several more [6]. They are known to serve many biological functions including control of chromosome dynamics, splicing, RNA editing, translation inhibition, and mRNA destruction [6]. ncRNAs can exist to perform generic housekeeping functions across multiple tissue types or they can be hyper-specific to tissues, diseases, and conditions. For example, there are several muscle-specific miRNAs that are ubiquitously expressed in all skeletal muscles (e.g., miR-1, miR-133, miR-206) while there are others that are only *significantly* expressed in dystrophic conditions (e.g., miR-146b, miR-221, miR-155, miR-214, and miR-222) [7].

**Fig. 1:**
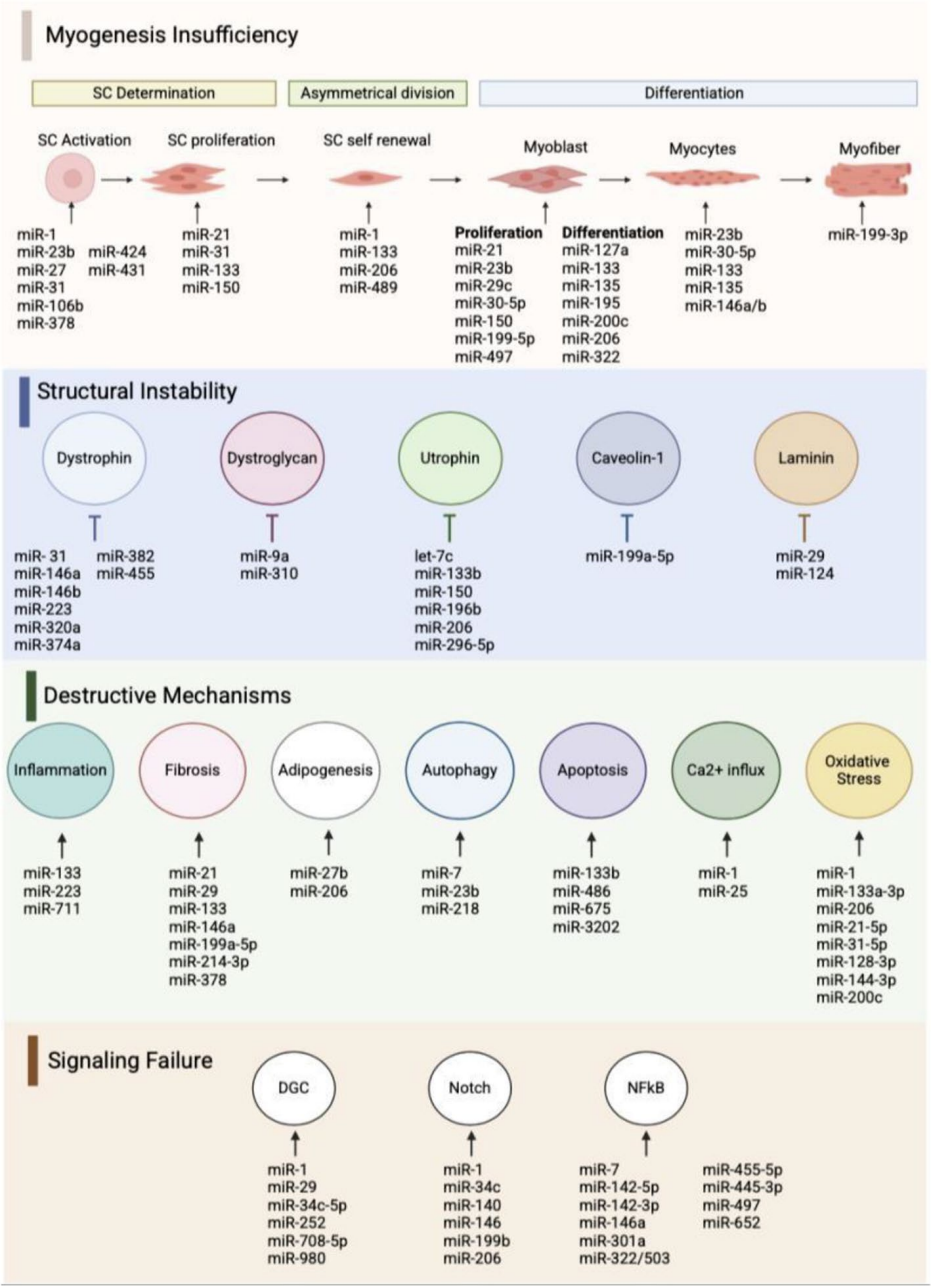
A schematic representation of the most prominent ncRNAs involved in major pathophysiological mechanisms in muscle dystrophy

In this article, we review all the most pertinent literature on the role of ncRNAs (particularly miRNAs) and their associated proteins in the pathogenesis of MD. The findings suggest that a key driving force of MD is the dysregulation of muscular structures and pathways that were previously functioning normally—a dysregulation that often stems from defective transcription factors and their associated ncRNAs. We attempt to delineate the common pathways and culprits shared by all MD subtypes as well as the specific influencers unique to each subtype. We classify the pathophysiologies of all MD subtypes into four main common themes: (1) insufficiency of myogenesis, (2) structural instability of the essential myofiber architecture, (3) mechanisms of degradation/wasting, and (4) failure of signaling pathways. Each of these four themes co-exist and influence one another in all MD subtypes to varying extents; this means that they may occur in different sequences, arrangements, durations, and impact levels. Figure 1 summarizes the most prominent ncRNAs involved in the pathological mechanisms of muscular dystrophy. This article aims to paint a cohesive picture of the connections between miRNAs and other ncRNAs, and their associated protein effectors in the context of these four pathological themes, as well as shed light on the gaps in literature that could be targeted in future research.

## Myogenesis insufficiency

One of the key premises underlying MD is that they occur secondary to malfunctions of initially healthy (or at the very least, functional) muscular tissue [8]. This is evidenced by the fact that the muscles of all MD patients start expressing dystrophic changes at variable life intervals ranging from infancy to adolescence. Interestingly, even with congenital MD (CMD), the muscles function normally during embryonic development. However, they become disturbed after birth, as dystrophic changes do not begin to appear until shortly afterward [9]. Generally, the muscle fibers differentiate, proliferate, and develop normally in the early years of life and only begin to exhibit dystrophic signs after a specific amount of time—some begin almost immediately after birth while others begin in the 3rd and 4th decades [10].

Therefore, this trend suggests that there are certain disease *initiators* and *drivers* involved in the process. It has been demonstrated in healthy muscle tissue that muscle satellite cells serve as potent myogenic progenitors and are able to maintain a functional population by self-renewal without the need for extrinsic stem cells [11].

On the one hand, in healthy muscles, the natural progression of ageing leads to a modestly decreased satellite cell pool, however the regenerative capacity remains relatively unchanged throughout the course of a lifetime [12]. On the other hand, in dystrophic muscles, researchers have speculated that an excessively depleted satellite cell pool due to constant need for regeneration may be the key culprit in myogenic failure. Nevertheless, this hypothesis has largely been put to rest as it has been shown that satellite cells of mice with muscular degeneration features retain the capacity to differentiate and proliferate when transplanted into healthy muscle tissue [13], thus implying that the pathological origin is intrinsic to the integrity of the stem cells relative to the host environment. In addition to being largely unchanged in MDs, the satellite cell pool was also reported to be elevated in some cases [14]. Therefore, this suggests an issue of *quality* rather than *quantity* of the myogenic program. The myogenic program can be categorized chronologically into satellite cell activation (from quiescence), proliferation, self-renewal, and differentiation. In this section, we attempt to describe the key ncRNAs and proteins involved in each stage that function normally under healthy muscle conditions, as well as how their perturbations contribute to the faulty myogenesis in MDs (Table 1).

**Table 1:** A summary of the most prominent ncRNAs and their direct targets in myogenesis insufficiency

ncRNA	**Target**	**Type of MD/cell**	**Pathological role**	**References**
miR-1	Pax7	C2C12	Myogenic differentiation	[15]
	HDAC4	Healthy human myoblasts	Myogenic differentiation	[16]
miR-17-5p	Jak1, Ccnd2, Rhoc	C2C12	Promote myogenic differentiation	[17]
miR-18a	Igf1	C2C12	Myotube atrophy Decreased phosphorylation of Akt & Foxo3	[18]
miR-21	YOD1 YAP	DMD	Hippo signaling Proliferation Cell survival	[19]
miR-23b	MBNL1/2	DM1	Myogenic differentiation	[20]
miR-26a	Smad1 Smad4	C2C12	Targets TGF-b/BMP signaling pathway Myogenic differentiation	[21]
miR-27	Pax3	MuSC	SC quiescence	[22]
miR-29	HDAC4	C2C12 Primary muscle cells	Myogenic differentiation	[23]
miR-29c	ASB2	DM1	Degradation of actin-binding proteins	[24]
miR-30-5p	MBNL1/2	DM1	Myogenic differentiation	[25]
miR-31	Myf5	MuSC	Myogenic differentiation	[26]
	Dystrophin	DMD BMD Myositis	Dystrophin translation	[27]
miR-106b	Myf5	MuSC Myoblast	Myogenic differentiation	[28]
miR-125b	IGF2	C2C12	Myogenic differentiation	[29]
miR-133	MAML1	DMD	Timing myogenic differentiation	[30]
	SRF	C2C12	Myoblast proliferation	[31]
miR-135	MEF2c	DMD	Timing myogenic differentiation	[30]
miR-146	Numb	C2C12	Inhibits Notch signaling Promotes myogenic differentiation	[32]
miR-146a/b	Dystrophin	DMD	Dystrophin translation Delayed myogenic termination	[33]
miR-148a	DMPK	DM1	Myogenic regeneration	[34]
miR-150	CDK3	DMD	Myoblast proliferation	[35]
miR-155	MEFA2A	C2C12 myoblast	Represses skeletal muscle differentiation	[36]
miR-199a-5p	FZD4, JAG1, WNT2 (WNT signaling)	DMD	Balances myogenic proliferation & differentiation	[37]
miR-200c	P66Shc	DMD	Inhibits myogenic differentiation	[38]
miR-206	Pax7	Primary isolated SC C2C12	Myogenic differentiation	[15]
	Twist-1	DM1	Myogenic differentiation	[39]
	DMPK	DM1	Myogenic regeneration	[34]
miR-218	MBNL1/2	DM1	Myogenetic differentiation	[20]
miR-221 miR-222	p27	C2C12	Myogenic differentiation	[40]
miR-223	IGF2	QM-7 cell line CPM	Inhibits myoblast proliferation	[41]
	ZEB1	QM-7 cell line CPM	Promotes myoblast differentiation	[41]
miR-322	Cdc25A	C2C12	Myogenic differentiation Promotes cell cycle quiescence	[42]
	SETD3	C2C12	Represses myogenic differentiation	[43]
	Celf1	DM1	MK-ERL signaling Myogenic differentiation	[44]
miR-378	MyoR	C2C12	Myogenic differentiation	[45]
	Igf1r	DMD	IGF1R signaling SC activation	[46]
miR-411	YAF2	FSHD	YY1 inhibition Myogenic differentiation	[47]
miR-424	Cdc25A	C2C12	Myogenic differentiation Promotes cell cycle quiescence	[42]
miR-431	Pax7	DMD	SC activation Myogenic differentiation	[48]
miR-486	Pax7	C2C12	SC activation Myogenic differentiation	[49] [50]
	Dock3 PTEN	DMD	PTEN/Akt signaling Sarcolemmal stability Myotube apoptosis/fusion	[51]
miR-503	Cdc25A	C2C12	Myogenic differentiation Promotes cell cycle quiescence	[42]
Lnc-31	Rock1 mRNA YB-1 protein	C2C12 Human myoblast	Promotes myoblast proliferation	[52]
Linc-MD1	miR-133 miR-135	DMD	Timing of myogenic differentiation	[30]
Malat 1	miR-133	C2C12	Modulate Srf expression Myoblast differentiation	[53]

*MuSC *muscle satellite cell, *CPM *chicken primary myoblast, *SC *satellite cell, *DMD *Duchenne muscular dystrophy, *BMD *Becker muscular dystrophy, *FSHD *facioscapulohumeral muscular dystrophy, *DM1 *myotonic muscular dystrophy type 1, *miR *microRNA, *LncRNA *long non-coding RNA

### All MDs

In the activation of quiescent MuSCs phase, there are several key ncRNAs that become perturbed or lead to activation issues. One of the most important ncRNAs that is significant in every phase of myogenesis is miR-31. Normally, miR-31 silences Myf5 mRNA activity by keeping it sequestered in messenger ribonucleoprotein (mRNP) granules, consequently maintaining quiescence and preventing MuSC activation until required [26]. When the muscle niche indicates a need for new muscle, Myf5 is released from the mRNP granules and enters polysomes where it undergoes translation to produce the proteins necessary for myogenesis [26]. miR-31 is particularly significant as it is upregulated in all subtypes of MD (with the exception of Calpain-3 disease models), and therefore contributes to impaired myogenesis in almost all forms of dystrophy [26].

There is growing evidence on Pitx2c and Pax3, both of which are notable transcription factors involved in proper muscle function, playing versatile roles in muscle fibers extending from embryonic development to adult muscle maintenance and repair. Pax3 is post-transcriptionally regulated by the snRNA U1 and miR-206; snRNA U1 controls the alternative polyadenylation of its transcript, which can affect the length of its 3’UTR thereby controlling its susceptibility to being targeted by miR-206 [54]. Interestingly, miR-206 is dysregulated in most MDs as will be discussed later. Moreover, several studies have leveraged this knowledge to induce regeneration of both MuSCs and myofibers in dystrophic mouse models via the introduction of PAX3-positive cell [55, 56]. MiR-27 is a downstream effector as Pitx2c regulates miR-27 and Pax3 expression, thereby maintaining MuSCs in a pre-differentiated state [22]. Pitx2c also downregulates miR-15b, miR-23b, miR-106b, and miR-503, inducing proliferation of myoblasts [28]. Pitx2 is particularly important to the understanding of MDs since its expression is maintained at high levels in dystrophic extra-ocular muscles (EOMs) but is low in dystrophic limb skeletal muscle [57], which suggests that it may play a role in the sparing of EOMs commonly observed in MDs [58].

During the differentiation stage, there are two key miRNAs involved in almost all MD subtypes: miR-155 and miR-206. miR-155 represses Mef2a, a differentiation inducer, thereby preventing differentiation [36]. miR-155 is known to be dysregulated in most of the dystrophic pathologies, but particularly in Duchenne muscular dystrophy (DMD), Becker muscular dystrophy (BMD), facioscapulohumeral muscular dystrophy (FSHD), limb–girdle muscular dystrophy 2 A (LGMD2A), LGMD2B, myotonic dystrophy (DM), and mitochondrial myopathy (MM) [59]. miR-206 is one of the most notorious muscle-enriched miRNAs (myomiRs) and is heavily involved in various stages of myogenesis [60]. miR-206 produces its effects on myoblast differentiation via influencing Pax3/Pax7, Cx43, HDAC4, nPTB/PTB2, DNA Pola1, Id1-3, and MyoR [60]. Besides downregulating Pax3 and Pax7 to transition from the proliferation phase to the differentiation phase, miR-206 also decreases the expression of Cx43 expression, hence reducing the communication between growing muscle fibers, which in turn leads to differentiation [60]. Similarly, it inhibits HDAC4 to promote differentiation [23], and regulates nPTB/PTB2 which has downstream effects on muscle differentiation [61]. Moreover, miR-206 negatively regulates translation of the DNA polymerase subunit Pola1, thereby promoting differentiation [62], and decreases expression of Id1-3 and musculin (MyoR) (inhibitors of myogenic transcription factors), ultimately facilitating differentiation [62]. Due to miR-206’s far-reaching influence over all these different myogenic pathways, it is no surprise that it is a key player in the myogenic impairment of most subtypes. Many studies have provided evidence of miR-206 as a reliable circulating biomarker of disease severity, with many showing that miR-206 dysregulation may directly contribute to myogenic failure. In contrast, others have claimed that miR-206 has a protective role in various MDs (e.g., inducing muscle regeneration, delaying progression of pathology) [39, 63–66].

### DMD

In the context of DMDs, miR-31 is markedly increased, and some investigators have suggested that these elevated levels are probably due to an “intensive regeneration” that occurs in DMD, possibly as an attempted compensation for the inability to complete myogenesis [27]. miR-31 also exerts its influence on dystrophin—a protein typically associated with muscle structure but now understood to contribute to other pathways, including myogenesis [67]. The investigators furthered this line of reasoning by demonstrating that miR-31 inhibition enhanced the outcome of exon skipping therapy by alleviating dystrophin translational repression and thus improving dystrophin production in human DMD myoblasts [27]. In another study, mdx mice were injected with satellite cells overexpressing Pitx2c and observed that miR-31 was significantly downregulated, and dystrophin expression was increased [68]. This further solidifies the role of miR-31 in quiescence disruption in DMD and also provides new evidence that miR-31 is regulated by Pitx2c [68]. In addition, miR-106b supports healthy muscle myogenesis by reinforcing Myf5 inhibition, hence contributing to the blocked activation of MuSCs—as such, miR-106b must be downregulated in order to activate these satellite cells [28]. However, a recent study has shown that miR-106b is actually overexpressed in dystrophic muscle stem cells and that injection of mdx mice with anti-miR-106b leads to muscle regeneration [69].

Following activation, the undifferentiated satellite cells go through an intermediary bifurcation stage where they are either primed into the proliferation stage or revert to quiescence (known as “self-renewal”). Significance of this perturbed process is evidenced by the reduced self-renewal potential of mdx mice [70], as well as the improvement of condition via targeting of self-renewal satellite cells in mdx mice [71]. Several myomiRs (including miR-1, miR-133, miR-206) are known to negatively regulate PAX7 protein expression directing the process of self-renewal, where its overexpression impairs self-renewal, and its inhibition enhances it [15, 72]. In DMD patients, serum samples have shown significant upregulation of miR-133 [73], which could suggest an influence on self-renewal potential. Additionally, miR-146, a known dystromiR, targets Numb which inhibits Notch signaling, and inhibition of this miRNA restores Numb expression thereby promoting MuSC differentiation [32]. However, miR-146 has been demonstrated to be dysregulated in DMD [59], and furthermore, inactivation of Numb in mdx mice promotes senescence of myogenic cells [74]. Collectively, this evidence suggests that even the MuSCs that manage to get activated are still at risk of impaired self-renewal.

After the division/self-renewal phase, the myoblasts that do not self-renew enter the proliferative phase. Among the most significant ncRNAs in this phase are miR-133, miR-150, Lnc-31, and miR-21. miR-133 has been shown to enhance myoblast proliferation by repressing serum response factor (SRF) [31]. Indeed, miR-133 is increased in DMD patients which may initially have positive effects on proliferation, but ultimately leads to dysregulation of proliferating myoblasts in DMD muscle [72, 75]. miR-150 is known to directly suppress the proliferation inhibitor CDK3 and hence increased expression of miR-150 in mdx mice is closely related to the decreased CDK3 levels observed [35]. Lnc-31 is also involved in proliferation issues of DMD myoblasts through its effects on myogenin and ROCK1. Lnc-31 sustains cell proliferation and counteracts differentiation by sequestering myogenin away from its target genes that promote differentiation [76]. Moreover, Lnc-31 upregulates ROCK1 in the proliferative state where ROCK1 exhibits differentiation-repressing activity [52]. Lnc-31 has been shown to be increased in both mdx mice and human DMD, and thus it follows that there would be increased Lnc-31-induced proliferation [76]; however, this may not be ideal as excessive or unnecessary proliferation could actually be a detriment to regular muscular development in DMD [77]. miR-21 is also implicated in defective proliferation via its effects on the Hippo Pathway [19]. miR-21 levels are elevated in DMD muscles, which reduces YOD1 and in turn increases LATS1/2 expression, thereby decreasing the pool of active YAP (which is itself a target of miR-21 as well) [19]. This cascade eventually leads to decreased expression of YAP-mediated target genes and ultimately attenuates proliferation and cell survival [19].

The last main stage of the myogenic program is differentiation, in which proliferating myoblasts differentiate into the various types of specialized muscle cells. miR-26a normally targets Smad1 and Smad4 to inhibit differentiation through their effects in the TGF-β/BMP pathway [21]. Moreover, investigators have shown that exogeneous miR-26a promotes myoblast differentiation while inhibition, via antisense oligonucleotides delays differentiation [21]. The dysregulation of miR-26a in DMD suggests possible perturbations of this pathway [59]. miR-29 expression leads to repression of HDAC4 translation—a known inhibitor of muscle differentiation [23]. It is highly likely that this pathway is disturbed in dystrophic muscle due to the apparent reduction of miR-29a levels in DMD muscle, the evidence that miR-29 downregulation results in impaired regeneration in mdx mice, and the enhanced regeneration upon miR-29 oligonucleotide injection in mdx mice [78, 79]. miR-155 represses the known differentiation inducer, Mef2a [36], but this miRNA is dysregulated in DMD [59] and is significantly increased in mdx mice [80]. miR-200c increases the phosphorylation of p66Shc thereby inhibiting differentiation, but this miRNA is significantly upregulated in mdx mice which could implicate its involvement in DMD’s impaired differentiation [81]. This is further evidenced by the fact that anti-miR-200c injection ameliorates myogenic differentiation [81]. miR-378 targets the myogenic repressor, MyoR, to mediate differentiation of myoblasts, however it is decreased in dystrophic muscles of animal models [45]. Furthermore, knock-out of miR-378 in mdx mice did not alter the *number* of myoblasts but did result in diminished contribution of activated myoblasts in the total myoblast pool providing proof of its role as a modulator of differentiation [46]. Upregulation of the lncRNA H19 is also involved by increasing the expression of miR-675, a miRNA processed from exon 1 of the H19 lncRNA, which in turn promotes myoblast differentiation [82]. Investigators have shown that both H19 and miR-675 are decreased in DMD and that increasing H19 and miR-675 levels via introduction of synthetic preimplantation factor (sPIF) promoted differentiation [83]. The authors observed increased myogenesis and decreased expression of fibrogenic markers (collagen I/II, TGF-β) and suggested that H19-induced miR-675 expression may inhibit TGF-β pathway (a pathway known to suppress myogenesis and promote fibrogenesis), thereby relieving inhibitory constraints on myoblast differentiation and favoring progression to myotubes. The study also reported a simultaneous reduction in let-7 through both transcriptional downregulation and H19-mediated sponging. Let-7 normally represses utrophin, a dystrophin homolog which supports sarcolemmal stability and myotube maturation, therefore its reduction removes this repression, allowing utrophin levels to rise. Together, H19-induced expression of miR-675 and suppression of let-7 may create a pro-differentiation environment that enhances myoblast fusion and maturation, particularly in DMD myoblasts where these pathways are impaired [83]. Another lncRNA, linc-MD1, is typically downregulated in DMD myoblasts and is known to sponge the differentiation-inducing miR-133 and miR-135 by competing with their targets MAML1 and MEF2c respectively in a time-dependent manner, and restoration of linc-MD1 partially regains the expression of these myogenic factors [30]. miR-431 regulates myogenic differentiation in C2C12 cells by modulating Pax7 expression, prompting investigators to test the role of miR-431 in dystrophic muscle [48]. They found that overexpression of miR-431 in mdx mice led to a reduced dystrophic phenotype due to enhancements of myoblast proliferation and differentiation (evidenced by the increased proliferating Pax7^+^/MyoD^−^ and differentiating Pax7^−^/MyoD^+^ myoblasts) [48].

Certain ncRNAs exert their influence on particular sub-stages of differentiation. For example, impaired miR-486 functionality seems to prevail in all sub-stages of differentiation. Normally, miR-486 kickstarts the differentiation stage by downregulating PAX7 [49], while in the later stages downregulates DOCK3 (a regulator of the AKT/PTEN pathway) to maintain the driving force of differentiation [51]. One study found that overexpression of miR-486 delayed muscle regeneration and influenced cell cycle kinetics through direct modulation of FOXO1 gene targets CDKN1 (p21) and CDKN1B (p27) of the Akt/PTEN pathway; however, miR-486 is decreased in DMD muscle and so it is unclear whether this particular pathway is prevalent in dystrophic muscle [84]. Furthermore, miR-486, miR-1, and miR-206 have been shown to demonstrate delayed induction in sphingosine phosphate lyase (SPL)-knockdown mice. SPL is involved in muscle regeneration through its regulation of S1P [50]. This study found that knocking out SPL led to decreased myomiR levels, increased PAX7, and impaired differentiation which was mediated by its receptor S1PR [50]. Finally, miR-199a and miR-221/222 are potentially involved in the defective differentiation observed in DMD. miR-199a, which is itself regulated by SRF, normally downregulates multiple differentiation regulators in the WNT signaling pathway to facilitate proper differentiation [37]. This process could possibly be negatively impacted in DMD as miR-199a is dysregulated in mdx mice, dystrophin-deficiency zebrafish, and dystrophin-deficient human muscle [37]. However, it remains unclear how miR-199a dysregulation might alter differentiation since overexpression of miR-199a in transgenic zebrafish muscle resulted in pathological effects (e.g., abnormal myofiber disruption, sarcolemmal membrane detachment) while administration of miR-199-3p mimics in mdx mice led to positive effects (e.g., improved muscle strength, decreased serum CK levels) [85]. The downregulation of miR-221/222 cluster upregulates p27 (cell-cycle inhibitor) initiating cell cycle arrest [40] (which is a prerequisite for differentiation [86])—and it has been shown that miR-222 is dysregulated in DMD [59]; however, it is currently unclear how this contributes to differentiation (i.e., as a form of compensation for impaired myogenesis versus direct causation of perturbed differentiation in DMD).

### BMD

BMD is very similar to DMD as it is caused by mutation of the same key gene (dystrophin) with very similar, albeit less severe, manifestations [87]. Therefore, it follows that it shares some, but not all, of the associated ncRNA and protein effectors involved in impaired myogenesis. The miR-31/Myf5 pathway is impaired in BMD as miR-31 is upregulated both in bmx mice (BMD mouse model) as well as BMD muscle biopsies [88, 89], implying improper activation signaling. Self-renewal of activated MuSCs may be potentially impaired as miR-133 (previously mentioned to increase Pax7 expression) is increased in BMD patient serum [64], although it is unclear whether this upregulation in serum is specifically associated with dysregulation in muscle. Furthermore, the only two miRNAs known to be involved in defective differentiation in BMD are miR-155 and miR-221, as both are dysregulated in BMD. However, miR-155 dysregulation was reported to be less significant in BMD patients than in the other subtypes measured [59], and miR-221 was reported to be significantly overexpressed in BMD patients [90].

### DM

DM is another prevalent form of MD caused by autosomal-dominant expanded CTG repeats in the DMPK gene and is associated with many ncRNAs [91]. Regarding proliferation, miR-223-5p inhibits IGF2 which blocks MuSC proliferation, meanwhile MyoD upregulates miR-223-3p which in turn inhibits proliferation [41]. miR-223 has been shown to be significantly downregulated in both the skeletal muscle and heart muscles of DM1 mice [92]. However, there is no current literature on this miRNA in humans, so it is unclear whether this downregulation is a compensatory mechanism for wasting muscle or if it itself contributes to disease pathology. On the other hand, circHIPK3, a circRNA commonly referenced in myogenesis literature [93], positively regulates human cell proliferation by sponging miR-29b and miR-193a [94], which are downregulated in DM1 and DM2 [95, 96], thus suggesting a potential mechanism for circHIPK3-associated myogenesis dysfunction.

There are several ncRNAs involved in perturbation of differentiation in DM, the most significant of which is the myomiR miR-1. miR-1 is directly involved in differentiation through its effects on HDAC4 and Pax7. Under normal conditions, miR-1 suppresses HDAC4 expression during differentiation to promote further pro-myogenic activity [16], but also suppresses Pax7 after differentiation has already begun to sustain the process [15]. However, miR-1 levels are downregulated in both Drosophila DM models as well as in DM1 patients [97, 98]. Moreover, it has been demonstrated that loss of miR-1 directly leads to inhibition of myoblast differentiation [15]. As such, it seems quite evident that miR-1 and its associated targets are heavily involved in the impaired differentiation in DM. miR-125b targets the differentiation inducer IGF2 thus facilitating myoblast differentiation [29]—but it has been shown that miR-125b is downregulated in the DM2 variant and therefore it possibly contributes to this pathological pathway, at least for this specific variant [96]. Moreover, miR-223 inhibits ZEB1 in differentiating myoblasts but is downregulated in DM1 mice models and therefore possibly contributes to perturbed differentiation [41]. Murine miR-322 (or its human orthologous miRNA equivalent, miR-424) contributes to differentiation through its influence over multiple proteins and coding sequences, but the literature seems to suggest contradictory roles. For example, miR-322/−424 (along with miR-503) induce muscle differentiation by downregulating Cdc25a [42]; however, miR-322 also represses muscle differentiation by inhibiting SETD3 [43]. Additionally, the introduction of ectopic miR-322 or miR-503 directly targets CUG repeats and Celf1 in DM1 cell model and rescues it from defective differentiation [99]. This is an important discovery as Celf1 (a cell cycle regulatory protein) is one of the main contributors to defective myoblast differentiation in DM1 [44] via the suppression of the Celf1-inducing MEK-ERK signaling pathway [100]. On a related note, Celf1 was shown to be downregulated in DM1 mouse models after introduction of miR-206 [101]. Investigators found that miR-206 induction both inhibited Celf1 and promoted MyoD expression, thereby partially rectifying the defective differentiation [101]. Twist1 is another differentiation-associated protein that involves the joint action of miR-206 and MyoD [39]. MyoD upregulates miR-206 which in turn inhibits Twist1, thereby inducing differentiation in both normal and DM1 cells [39]. The investigators suggest that this pathway exists to rescue defective differentiation rather than contribute to it [39]. miR-206 and miR-148a have been shown to directly target DMPK 3’UTR transcripts in a synergistic manner leading to decreased DMPK protein expression [34]. This is significant as previous reports have shown that decreased DMPK levels delay differentiation in myotonic myoblasts [102]. miR-30a-5p, miR-30b-5p, and miR-30e-5p were shown to regulate two key MBNL1 protein isoforms, and miR-30-5p in particular affected MBNL1 splicing to ultimately lead to inhibition of myogenic differentiation [25]. Additionally, miR-23b and miR-218 (which are upregulated in DM1 myoblasts) have been directly shown to suppress MBNL1 and MBNL2, and silencing of these miRNAs using antagomiRs greatly improved DM1 mouse model phenotypes [20]—although a definitive connection between these miRNAs and improved myogenic processes has yet to be established. miR-29c, another dysregulated miRNA in DM1, was shown to directly inhibit ASB2, which could potentially affect differentiation as this protein was previously tied to defective myogenesis via its destruction of Filamin B [24]. Finally, the aforementioned miR-155-Mef2a axis may also be involved in DM differentiation impairment as it is significantly dysregulated in DM patients [36, 59].

### FSHD

The dysregulations in FSHD’s myogenic program bear some resemblance to those of the previously mentioned MD subtypes but are also unique in other ways. The previously mentioned faulty pathways are the miR-31-Myf5 [26], miR-1-HDAC4 [16] and miR-1-Pax7 [15], and miR-155-Mef2a [36] axes. Starting with miR-31-Myf5, miR-31 has been shown to be increased in DUX4-induced FSHD-like mouse models as well as in FSHD patient serum [65], implying that it is involved in repressing activation of MuSCs by sustained sequestration of Myf5. The key differentiation actor, miR-1, is downregulated in FSHD patients and therefore it is unlikely that its pro-differentiation trajectories are maintained in this patient population [103]. miR-155 has been shown to be dysregulated in FSHD patients and so it probably cannot effectively regulate differentiation via Mef2a [59]. On the other hand, the myomiRs are upregulated by DUX4, and both DUX4 and the myomiRs have been shown to be highly expressed in FSHD myoblasts [104]. However, induced overexpression of the myomiRs in FSHD myoblasts did not prematurely enter the myogenic differentiation program, which is at odds with the implication that myomiR presence should hasten the process [104]. Conversely, DUX4’s involvement in the overexpression of cell cycle and DNA-damage related genes was observed [104]. As such, researchers hypothesized that defective myogenesis in FSHD occurs as a result of two competing biological programs: i.e., DUX4-induced overexpression of myomiRs which drives differentiation forward versus concomitant overexpression of cell cycle genes and DNA damage-related genes which hinder the progress [104].

miR-18a normally regulates proliferation processes by decreasing Igf1 and it has been shown that overexpression of miR-18a increases expression of MuRF1, Atrogin-1, and CTSL (all of which are involved in the muscle atrophy process) [18]. This miRNA has been shown to be significantly dysregulated in a few MDs but particularly in FSHD, and so it follows that it may not effectively regulate proliferation in FSHD [59]. After proliferation, there are several miRNAs that contribute to defective differentiation. miR-17 acts on the JAK1–STAT1–STAT3 cascade to cease proliferation and drive differentiation [17], and miR-17-5p is significantly dysregulated in FSHD [59]. However, further proof of miR-17’s role in FSHD myogenesis impairment is provided by its association with miR-19. miR-19 is known to reverse cell death caused by miR-17 and simultaneous administration of both can promote differentiation [17]; however, miR-19 is differentially expressed in FSHD muscle and therefore seems unlikely to serve in this compensatory role [105]. miR-100 upregulates myogenin and α-actin genes thereby accelerating the differentiation process [106], but in FSHD muscle this miRNA is downregulated and so may not aid in the much-needed acceleration of myogenesis in wasting muscle [104]. Finally, miR-411 inhibits YAF2 which in turn suppresses MyoD and myogenin, both of which are key differentiation regulators [47]. This miRNA is upregulated in both primary and immortalized FSHD myoblasts and therefore is likely to further suppress these myogenic regulators even more than it already does in healthy muscle [47].

### LGMD

Similar to FSHD, LGMD shares some dysregulated axes such as the miR-18a-Fgf1, miR-1-HDAC4 and miR-1-Pax7, and miR-17-JAK-STAT axes. miR-18a is dysregulated in both LGMDA2A and LGMD2B and so is unlikely to regulate proliferation for these two LGMD variants [59]. miR-1 is also decreased in the muscle of LGMD murine models and therefore is unlikely to suppress or sustain differentiation [107]. miR-17-5p is dysregulated in LGMD as well and so probably does not drive differentiation forward [59].

Moreover, there are two possible miRNAs [108] unique to the differentiation impairment in LGMD: miR-22 and miR-431. miR-22 inhibits HDAC4 thereby increasing its downstream target MEF2C and ultimately promoting differentiation. miR-22 is increased in Sgca-null mice (LGMD2D model) which could indicate that miR-22 promotes differentiation in a downstream pathway [109]; however, it is unclear whether that upregulation is a result of a feedback loop from dystrophizing muscle that signals for compensatory myogenesis or rather if it is itself a pathological mechanism (e.g., precocious/unwanted differentiation). In healthy muscle, miR-431 is downregulated by myostatin to suppress myoblast differentiation [108]. Currently, there are no studies on miR-431 levels in LGMD; however, it has been demonstrated that overexpression of miR-431 attenuates myostatin-induced inhibition of differentiation, and additionally, myostatin inhibition (via anti-myostatin antibodies) in δ-sarcoglycan murine model of LGMD2C/LGMDR5 leads to increased muscle mass in young mice [110]—collectively suggesting roles for miR-431-MSTN as pathophysiological pathways in LGMD.

### OPMD

In the context of oculopharyngeal muscular dystrophy (OPMD), there are three miRNAs that could possibly be implicated in defective myogenesis: miR-200c, miR-431, and let-7. As discussed earlier, miR-200c increases phosphorylation of p66Shc thus inhibiting myogenic differentiation, and recent reports have indicated that this miRNA is increased in both the muscle and saliva of OPMD patients [81, 111]. miR-431 is downregulated by myostatin to suppress myoblast differentiation, and injection of anti-myosin antibodies into the A17 mouse model of OPMD leads to increased muscle mass in young mice [108, 112]. Interestingly, the involvement of let-7 in impaired myogenesis is unique to OPMD. Let-7 has been shown to induce differentiation in *Caenorhabditis* *elegans* through its effects on PABPN1 [113] (which contains the GCG trinucleotide sequence that causes OPMD [114]). A study has provided key evidence that let-7 is involved in the regeneration and degeneration mechanisms of the OPMD myogenic program due to its significantly increased levels (which they suggest is a result of mutated PABPN1), as well as the unusually high percentage of Pax7-positive satellite cells in OPMD muscles [115]. It is still unclear exactly how these two entities stack hierarchically, but Hurschler et al. suggest that at least one of the associated transcription factors, PABP-2, acts either downstream or in parallel to let-7 in the heterochronic pathway [113].

## Structural instability

Skeletal muscle is composed of diverse protein groups with varied functions and contributions. The muscle apparatus consists of thick and thin filaments which slide over each other during contraction and relaxation—this is mostly executed by the actions of “actin” and “myosin” [116]. However, most MD subtypes are caused by perturbations of sarcolemmal and sub-sarcolemmal proteins located inside the myofilaments [117]. The main proteins involved are dystrophin, dystroglycan, dysferlin, laminins, lamins, emerin, sarcoglycans, dystrobrevins, and calpains. In this section, we describe how ncRNAs influence the pathophysiologies of MD subtypes (Table 2).

**Table 2:** A summary of the most prominent ncRNAs and their direct targets in structural instability

ncRNA	Target	Type of MD/cell	Pathological role	Reference
miR-9a	Dystroglycan	Drosophila MD model	ECM deposition in myotendinous junction	[118]
miR-31	Dystrophin	DMD C2C12 BMD GRMD	Decreases dystrophin expression Reduces exon skipping effectivity	[27] [88]
miR-124	Laminin γ1	HH27-28 spinal cords	Neuronal differentiation	[119]
miR-133	Utrophin	C2C12	Utrophin expression	[120]
	RhoA Cdc42	Human embryos & fetuses	Cardiac hypertrophy	[121]
miR-146a/b	Dystrophin	DMD C2C12 BMD GRMD	Decreases dystrophin expression Reduces exon skipping effectivity	[33]
miR-150	Utrophin	C2C12	Utrophin expression	[120]
miR-196b	Utrophin	C2C12	Utrophin expression	[120]
miR-206	Utrophin	DMD C2C12	Utrophin expression	[120]
miR-208a	THRAP1 Myostatin	Neonatal rat Cardiomyocyte Male C57BL/6	Regulates cardiac hypertrophy	[122]
miR-223	Dystrophin	DMD C2C12 BMD GRMD	Decreases dystrophin expression Reduces exon skipping effectivity	[33]
miR-296-5p	Utrophin	C2C12	Utrophin expression	[120]
miR-310	Dystroglycan	Drosophila MD model	Neuronal cell adhesion	[118]
miR-320a	Dystrophin	DMD C2C12 BMD GRMD	Decreases dystrophin expression Reduces exon skipping effectivity	[33]
miR-374a	Dystrophin	DMD C2C12 BMD GRMD	Decreases dystrophin expression Reduces exon skipping effectivity	[33]
miR-382	Dystrophin	DMD C2C12 BMD GRMD	Decreases dystrophin expression Reduces exon skipping effectivity	[33]
miR-675-3p/5p	Laminin, Desmin	LncRNA H19-deficient C2C12	Myogenic differentiation	[123]
Let-7c	Utrophin	DMD C2C12	Utrophin expression	[120]

*miR *microRNA, *DMD *Duchenne muscular dystrophy, *BMD *Becker muscular dystrophy, *GRMD *Golden retriever muscle dystrophy model, *MD *muscle dystrophy, *LncRNA *long non-coding RNA, *ECM *extracellular matrix

### Dystrophinopathies

Dystrophinopathies are a subset of MDs that are caused by decreased or absent dystrophin protein secondary to mutations of the X-linked dystrophin gene, namely DMD, BMD, and X-linked dilated cardiomyopathy (DCM) [124]. In this section, we delve deep into the pathophysiology of dystrophin dysfunction, the aberrant mechanisms of other associated proteins, and the influence of ncRNAs.

**Fig. 2:**
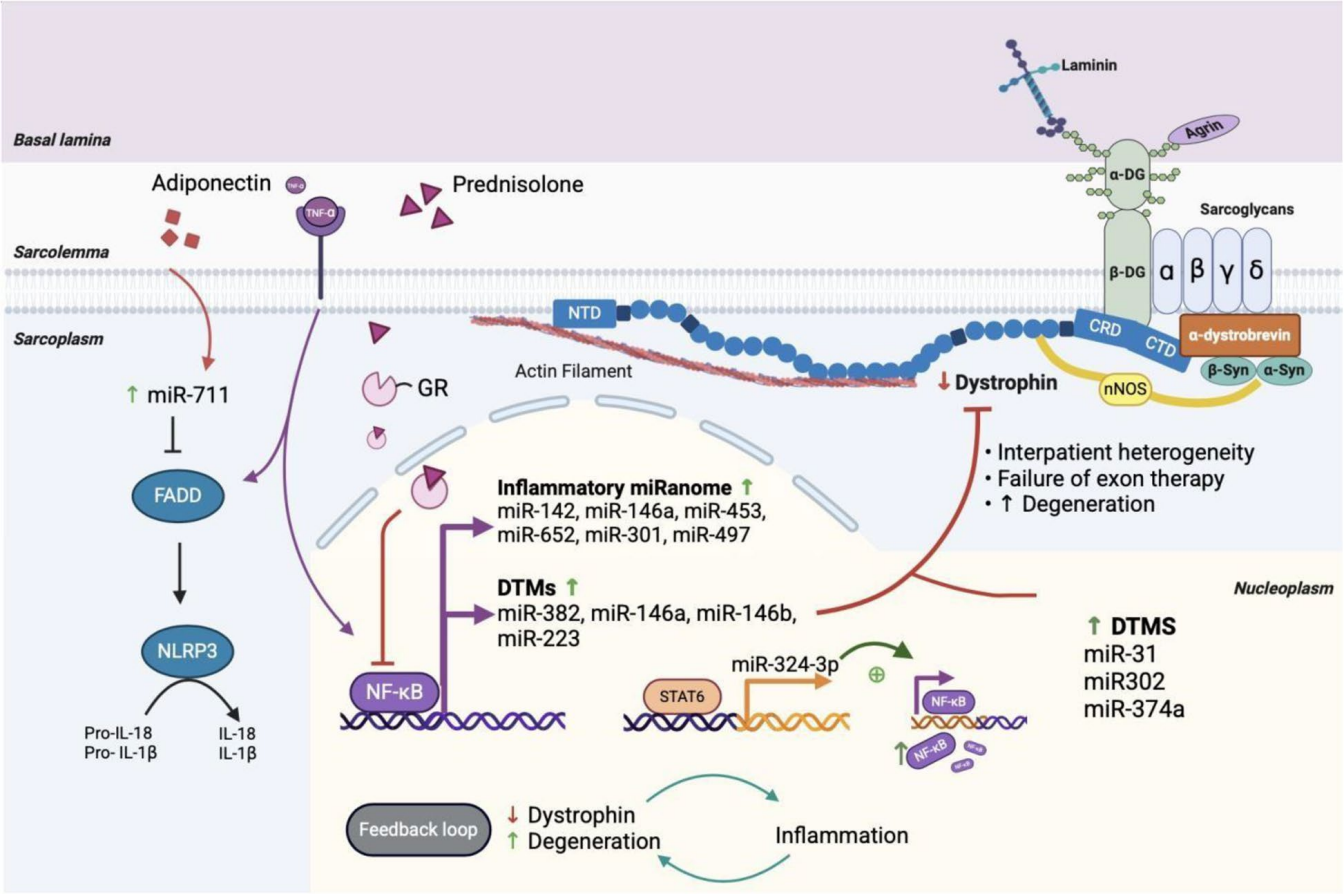
A regulatory feedback loop involving dystrophin deficiency, bouts of degeneration and inflammation driven by NF-κB-mediated miRNAs that modulate dystrophin expression. NF-κB stimulate two sets of miRs —inflammatory miRanome and DTMs— that down-regulate dystrophin protein expression. Dystrophin deficiency augments the degenerative process, contribute to dystrophin protein levels discrepancies, and mitigate exon therapy efficacy. Prednisolone, a corticosteroid used in DMD management, compromises NF-κB mediated miRNAs sequel. Abbreviations: *GR* glucocorticoid receptor, *FADD* FAS-associated death domain, *NF-κB* nuclear factor kappa-light-chain-enhancer of activated B cells, *STAT6 *signal transducer and activator of transcription 6, *α-DG* alpha-dystroglycan, *β-DG* beta-dystroglycan, *β-Syn* beta-syntrophin, *α-Syn* alpha-syntrophin, *nNOS* neuronal nitric oxide synthase, *IL-18* interleukin-18, *IL-1β* interleukin-1 beta, *DTMs* dystrophin-targeting microRNAs, *CTD* carboxy-terminal domain, *CRD* cysteine rich domain, *NTD* amino-terminal domain, *TNF-α* tumor necrosis factor alpha

DMD is the most devastating type among dystrophinopathies and therefore the literature on its associated ncRNAs and proteins is the most abundant. There are several key miRNAs identified as contributors to dystrophin’s degradation, referred to as the dystrophin-targeting miRNAs (DTMs) [33]. Fiorillo et al. showed that several DTMs (miR-146-5p, miR-382, miR-758, miR-214, and miR-494) either directly target or correlate with dystrophin levels, which lead to its downregulation and eventual functional failure [33]. It is hypothesized that the “normal” role of these DTMs in healthy muscle is to transiently reduce dystrophin expression to foster the proper microenvironment for remodeling myofibers [33, 125, 126]. However, pro-inflammatory cytokines (e.g., TNF-α) and pro-inflammatory signaling pathways (e.g., NFκB signaling), which are prevalent in dystrophinopathies, could possibly sustain the DTMs’ dystrophin inhibition longer than what would be necessary for myofiber remodeling, thus explaining a significant element of its pathophysiology [33]. Indeed, Fiorillo et al. provided ample evidence from mdx mice to back this hypothesis by showing that TNF-α treatment increased at least two DTMs (miR-146a and miR-223) and that pre-treatment with NFκB-inhibiting drugs, prednisolone and VBP15, suppressed DTM induction [33]. Furthermore, they showed that DTM upregulation is also age-dependent as it increased with age in both golden retriever muscular dystrophy (GRMD) and mdx muscle [33]. Importantly, other researchers have also highlighted the potential involvement of DTMs in the significant inter-patient and intra-patient dystrophin level discrepancies in DMD and BMD [27] (Fig. 2). Another miRNA that targets dystrophin but that is not classically referred to as a DTM, is miR-31. miR-31 directly promotes dystrophin mRNA degradation by targeting the 3′UTR of the gene, which ultimately blocks dystrophin translation [27]. Moreover, miR-31 has been shown to be widely abundant in DMD muscle biopsies compared to healthy muscle [27]. This is further compounded by the fact that exon-skipping therapeutic effects are accentuated when inhibiting miR-31 which in turn increases dystrophin rescue [27].

Besides dystrophin, there is some evidence that other structural proteins may also be perturbed in DMD. miR-206 overexpression has been shown to enhance β-dystroglycan (β-DG) expression in mdx mice [127]. This relationship is particularly interesting as miR-206 is typically upregulated in DMD [73], but DG loss is nonetheless still evident in DMD muscle [128], suggesting unsuccessful compensatory efforts mirroring the same failed attempts at compensation by miR-206 seen in impaired myogenesis. On the other hand, loss of dystrophin leads to a significant decrease in DG expression at the myofiber membrane, seemingly suggesting that DG structural impairment is directly associated with dystrophin perturbation in DMD regardless of the influence of miR-206 [128, 129].

The other main structure-related protein that may be involved in DMD pathology is caveolin-1. Caveolin-1 is a cell-surface protein expressed in most tissues, which is required to form specialized membrane curvatures used for various structural purposes (e.g., caveolae in skeletal muscle plasma membranes) [130, 131]. Although not necessarily a sarcolemmal protein, it is a key effector in the interface between the extra-cellular matrix (ECM) and the cytoskeleton [132], and in DMD, it has been shown that miR-199a-5p downregulates caveolin-1 expression, which may be a significant cause of the ECM dysregulation observed in DMD muscle [133, 134].

As BMD shares a very similar pathophysiology with DMD, it is no surprise that several influencing miRNAs are common between them as well. The DTMs have been shown to be elevated in BMD muscle, although this upregulation was more prevalent in BMD muscle lacking dystrophin compared to BMD with relatively higher levels of dystrophin [33]. The level of miR-31, which directly represses dystrophin translation, was shown to be slightly higher than in healthy controls, but significantly less than that of DMD, raising the question of its involvement in the pathophysiology of BMD [27]. Unlike DMD, there is currently no literature discussing the role of other ncRNAs or other structural proteins in BMD pathology.

DCM is a common later-stage feature of all dystrophinopathies and is the most common cause of death in this patient population [135]. Nevertheless, it is a pathology of an entirely separate tissue from skeletal muscle and therefore possesses unique pathophysiological mechanisms and characteristics. For example, reduced expression of miR-1 potentially leads to anti-hypertrophic outcomes in disturbed mechanical remodeling observed in several mouse models, but overexpression of this miRNA was shown to attenuate cardiac hypertrophy both in vitro and in vivo [121, 136]. miR-133 overexpression suppresses cardiomyocyte hypertrophy, while its inhibition induces cardiac hypertrophy via actions on cytoskeletal and myofibrillar rearrangement-related proteins (e.g., RhoA and CDC42) [121]. As mentioned previously, the myomiRs (including miR-1 and miR-133) are significantly dysregulated in DMD and BMD, and so the likelihood of them contributing to DCM in dystrophinopathies is very probable. miR-208a, a heart-enriched miRNA, is also possibly involved as it upregulates several cardiac hypertrophy-related genes (e.g., THRAP1 and myostatin) [122]. Furthermore, miR-208a has been reported to be a highly sensitive and specific biomarker in patients with heart failure with reduced ejection fraction (HFrEF), a known outcome of DCM in dystrophinopathies [137]. Finally, miR-339-5p is upregulated and released by exosomes from DMD patient-induced pluripotent stem cell-derived cardiomyocytes (DMD-iCMs) but induced downregulation of this miRNA leads to reduced cardiomyocyte death, thereby suggesting an important role in DMD-associated cardiac pathology [138].

Any discussion of the proteins involved in dystrophinopathies must include the influence of utrophin. Utrophin is an autosomally encoded protein that is homologous to dystrophin both in structure and in function [139]. It is typically found in the neuromuscular junction but in DMD/BMD, it is transported to the sarcolemma where it potentially serves a compensatory role by stabilizing the dystroglycan complex to the sarcolemmal membrane as dystrophin normally would [139, 140]. A study found six main miRNAs (let-7c, miR-150, miR-196b, miR-296-5p, miR-133b, miR-206) that target the utrophin 3′UTR in C2C12 mouse myoblast cell line [120]. Additionally, they found that inhibition of let-7c, miR-150, miR-196b, and miR-206 resulted in significant utrophin upregulation (while the reason behind miR-296-5p and miR-133b’s inability to do so remains unclear) [120]. Interestingly however, miR-206-KO (knock out) did not lead to any differences in utrophin expression compared to wild-type cells and miR-206 upregulation did impinge utrophin expression in mdx mice [66, 141]. On the other hand, there is compelling evidence that let-7 is indeed directly involved in utrophin expression as its inhibition in both C2C12 cells as well as dystrophic myoblasts led to increased utrophin expression [83, 120]. Nevertheless, even though utrophin may provide partial compensation, it is not enough to counteract the devastation of dystrophin loss or truncation in human dystrophic muscle [142].

### Dystroglycanopathies

Dystroglycanopathies are a group of pathologies caused by aberrant glycosylation of the structural protein DG (specifically, α-DG) [143]. This collection comprises several pathologies, including many CMD subtypes (e.g., Fukuyama congenital muscular dystrophy (FCMD), muscle-eye-brain diseases (MEB), and Walker–Warburg syndrome (WWS)), as well as several subtypes of LGMDs [143]. The dystrophin glycoprotein complex (DGC) plays a vital role in sarcolemmal membrane stability [144], and therefore any perturbation of this complex may lead to devastating structural impairments.

There are various ncRNAs, such as miR-9a, miR-137, miR-252, miR-310 s, miR-927, miR-959, miR-966, and miR-1000, that have been shown to contribute to DGC regulation in muscles of healthy animal models [118]. All of these miRNAs (except for miR-927) directly upregulate DG mRNA levels in Drosophila flies, and animal models bred to lack some of these miRNAs (miR-137, miR-927, and miR-966 in particular) exhibited severely compromised muscle integrity [118]. Meanwhile, overexpression of miR-137 and miR-927 during development caused lethality at various developmental stages of flies, while overexpression of miR-927 and miR-966 in adult flies caused increases in muscle degeneration [118]. Additionally, miR-9a and miR-310 s have been shown to maintain the appropriate levels of DG (required to canalize myotendinous junction formation) [118] and to buffer MD-related Type II Lissencephaly phenotype [145] (also known as Cobblestone Lissencephaly; a pathological manifestation observed in several dystroglycanopathies [146]).

Several variants of CMD are caused by generalized hypoglycosylation of α-DG while others are caused by the specific aberrant O-mannosylation of α-DG, the result of all being dysregulated or reduced α-DG which leads to dystroglycanopathy [147, 148]. Regarding LGMD, there are several subtypes directly associated with DG perturbation, the most significant of which is LGMD2I caused by FKRP-induced defective glycosylation of α-DG [149]. The miRNAs mentioned previously (miR-9a, miR-137, miR-252, miR-310 s, miR-927, miR-959, miR-966, and miR-1000) are all viable candidates for potential influencers of DG-related CMD and LGMD pathologies, but unfortunately there is currently no literature on their levels or activity in humans. However, α-DG hypoglycosylation significantly reduces its binding affinity for ECM components such as laminin (which in turn disrupts the DG-ECM linkage and leads to membrane fragility) [147], and laminin dysregulation in CMD *is* well-studied. As such, the relevant subtypes will be discussed in the “Laminopathies” section.

### Laminopathies

The most common form of laminopathy is merosin-deficient congenital muscular dystrophy (MDC1A) and it is caused by mutations in the laminin α2-chain gene (LAMA2) [150]. There are four main miRNAs related to the pathophysiology of MDC1A: miR-675, miR-29, miR-124, and miR-486. miR-675 is known to be differentially expressed in the dy2J/dy2J mice, a mouse model for MDC1A [151]. Interestingly, both miR-675-3p and miR-675-5p are processed from exon 1 of the lncRNA H19, and both miRNAs are upregulated during skeletal muscle differentiation. H19-deficient mice display abnormal skeletal muscle regeneration after injury, and it has been demonstrated that reintroduction of miR-675-3p and miR-675-5p by intramuscular injection can reverse the muscle defect observed via upregulation of laminin and desmin [123]. miR-29 and miR-124 have been identified as upstream regulators of laminin γ1 [119, 152]. Notably, these two miRNAs are known to regulate several effectors of muscle structure such as cytoskeletal regulatory proteins, cell–matrix adhesion molecules, and ECM proteins [153]. Moreover, overexpression of miR-29 in murine models leads to a myopathy that resembles Ullrich congenital muscular dystrophy [154]. Finally, the myostatin-miR-486 axis is not directly laminin-related, but there is a strong correlation between myostatin’s influence over miR-486 and miR-486’s role in CMD. miR-486 has been shown to be involved in several pathological processes in LAMA2-CMD and it is suggested that myostatin’s influence over miR-486 to regulate the IGF-1/Akt/mTOR pathway may be one of them [151]. This line of reasoning is further consolidated by two main insights: induction of IGF-1 signaling partially compensates for structural deficiency in dy2J/dy2J mice and myostatin elimination leads to a small but significant improvement in muscle status of dy2J/dy2J mice [155, 156].

### Others

There are several other disease clusters, which comprise many MD subtypes, centered on the notion of a perturbed muscle structural protein (e.g., sarcoglycanopathy, dystrobrevinopathy, calpainopathy, laminopathy), but unfortunately there is not enough information about the ncRNAs involved in their pathological mechanisms. However, some findings are starting to gain some momentum. For example, it is well established that calpain-3 and dysferlin are involved in membrane repair after sarcolemmal damage. Dysferlin deficiency in LGMD2B could possibly negatively impact calpain-3′s repair capacity, which in turn leads to an overall deterioration of the repair processes and the sustained accumulation of sarcolemmal damage observed in LGMD [157, 158]. Notably, several miRs have been identified as potentially contributory to this process including miR-519e, miR-154, miR-184, miR-485-3p, miR-654 [158], miR-301, and the DTMs [59]. Moreover, AAV-based miR-669a overexpression led to reduced hypertrophy of dystrophic myocardium and ameliorated the structure of DCM hearts in sarcoglycan (SG)-deficient (Sgcb-null) mice [159]. In conclusion, these findings demonstrate the significance of ncRNA-influenced structural instabilities in MD pathophysiology as well as the need to perform further research in this field.

## Destructive mechanisms

The most evident clinical manifestation of MDs by and large is the wasting of specific muscle groups, depending on the subtype. The dystrophic phenotype occurs as a result of several parallel mechanisms working in tangent. These mechanisms include persistent inflammation, fatty degeneration, autophagy, apoptosis, calcium dysregulation, oxidative stress, fibrosis, and more [160]. Different subtypes seem to correspond to different destructive phenotypes or “profiles.” For example, dysfunctional calcium influx seems to be a staple of calpainopathies, while DM muscle seems to exhibit significant signs of autophagy and apoptosis. On the other hand, there are certain mechanisms, and even specific pathways, that are universal to almost all MD subtypes (e.g., TNF- or inflammasome-mediated inflammation). This section attempts to describe and categorize all the key destructive mechanisms involved in MDs, as well as the ncRNAs and proteins involved in these pathological processes (Table 3).

**Table 3:** A summary of the most prominent ncRNAs and their direct targets in destructive mechanisms

ncRNA	Target	Type of MD/cell	Pathological role	Reference
miR-1	BAF60a BAF60b	C57Bl10 mdx mice FAP cells	Preferential increase in BAF60c FAP cell differentiation into a promyogenic phenotype	[161]
	G6PD	DMD	Oxidative damage	[162]
	B56α	Adult rat ventricular myocytes	Increases calcium release via RyR2 phosphorylation	[163]
miR-21	PTEN	DMD	AKT, ERK signaling Collagen deposition	[164] [165]
	COL1A1, COL1A2	DMD	Myogenic differentiation Fibrogenesis	[78]
miR-23b	MBNL1/2	DM1	Myogenic differentiation	[20]
miR-25	SERCA2a	H9c2 myoblast Murine heart failure model	Enhances cardiac dysfunction and fibrosis	[166]
miR-29a/b/c	COL1A3 FBN1 YY1	DMD	ECM accumulation Collagen deposition Impaired myogenesis	[79]
miR-133	BAF60a BAF60b	C57Bl10 mdx mice FAP cells	FAP cell differentiation into a promyogenic phenotype	[161]
	LASP1	Human mesangial cells Lupus nephritis	Promotes apoptosis	[167]
miR-206	BAF60a BAF60b	C57Bl10 mdx mice FAP cells	FAP cell differentiation into a promyogenic phenotype	[161]
	Runx1 (transcription factor)	FAPs (C57BL6 mice)	FAP cell differentiation	[168]
miR-214-3p	FGFR1	DMD	Fibrogenesis	[169]
miR-218	MBNL1/2	DM1	Myogenic differentiation	[20]
miR-223	IKKa	U937 Monocytes	Anti-inflammatory role	[170]
miR-378	FGF1	DMD	Fibrogenesis	[46]
miR-675	DUX4	FSHD	Apoptotic activity	[171]
	TGF-b	DMD	Myogenic differentiation Fibrogenesis	[83]
miR-711	NLRP3 Inflammasome	DMD	Inflammation and degeneration	[172]
miR-3202	FAIM2	FSHD	Apoptotic activity	[173]
LncRNA H19	miR-675	FSHD	Apoptotic activity	[82]

*miR* microRNA, *LncRNA* long non-coding RNA, *FAP* fibroadipogenic progenitor cells, *DMD* Duchenne muscular dystrophy, *DM1* myotonic muscular dystrophy type 1, *FSHD* facioscapulohumeral muscle dystrophy, *RyR2* ryanodine receptor, *DAPC* dystrophin associated protein complex, *ECM* extracellular matrix, *MD* muscle dystrophy

### Inflammation

Inflammation is a hallmark characteristic of emerging disease in almost every system affected by a short or long-term pathology [174]. Unsurprisingly, inflammation is a key player in the pathogenesis and disease progression of most MDs including DMD [175], BMD [176], LGMD [177], FSHD [178], DM [179], and CMD [180]. For long, the research consensus was that inflammation was merely an “epiphenomenon” that occurred in parallel to disease mechanisms in a non-specific manner; however, there is now a growing body of evidence suggesting that inflammation is not only specific but is also involved in several phases of the disease course [181, 182]. For example, it has been shown that complement proteins are activated *before* the onset of fiber necrosis in several MDs (e.g., FSHD, LGMD, CMD) and it is suggested that they may contribute to the subsequent necrotic process [183]. To add to that, damaged myofibers from several MDs (e.g., DMD, FSHD, dysferlinopathies, calpainopathies) express MHC-I on their surface, thereby implying inevitable interactions with inflammatory cells such as T cells and macrophages [184]. Moreover, an overexpression and prevalence of cytokines have been reported in several MDs (e.g., DMD, DM, LGMD, FSHD) and could be potential culprits in the muscle damage that occurs [185–187]. Inflammation, especially chronic inflammation, seems to be a common theme among most MDs. As such, it is important to note that inflammatory effectors not only directly damage muscle fibers, but also contribute to downstream mechanisms which lead to further damage (e.g., fibrosis, necrosis, impaired regeneration) [188, 189].

The NLRP3 inflammasome is one of the key effectors of inflammation in MD muscles. It is a collection of cytosolic receptors of the innate immune system involved in activating and promoting inflammatory components, including interleukin-1 (IL-1), IL-18, caspase-1, toll-like receptors (TLRs), and inflammation-promoting pathways (e.g., NF-kB) [190], that ultimately lead to muscle fiber destruction [191]. In the context of MDs, various downstream effectors of the cascade are found to be significantly dysregulated, including ASC protein, caspase-1, IL-1, and several TLRs [172]. Moreover, deletion of NLRP3 inhibited inflammation-mediated muscular atrophy [192], and studies suggest that glucocorticoid treatment of MD patients may inhibit NLRP3 expression and, in turn, reduce related pro-inflammatory cytokines (such as is observed in similar neuromuscular disorders [193]) [194]; these pieces of evidence further consolidate the notion that NLRP3 is directly involved in inflammation-mediated damage of muscles in MDs.

The NLRP3 inflammasome in muscles is also affected by several ncRNAs, the most prominent of which are miRNAs miR-133, miR-223, and miR-711, and the lncRNA MALAT1. miR-133a-1 has been shown to directly suppress the NLRP3 inflammasome and decrease the expression of several downstream inflammatory effectors (e.g., IL-1, IL-18) in other non-dystrophic inflammatory conditions [195]. For example, since MALAT1 has been shown to sponge miR-133 in the context of differentiation [53], it is also possible that it can contribute to the inflammasome expression. On the other hand, the contribution of miR-223 to this pathway is more clear-cut. miR-223-3p is an inflammatory miRNA involved in promoting skeletal muscle regeneration by regulating pro- and anti-inflammatory signals [196]. For example, miR-223 inhibits IKK-α in macrophages which, in turn, inhibits the activation of the NF-κB inflammatory pathway and ultimately reduces the production of pro-inflammatory cytokines [170]. In the context of MDs, miR-223 has been shown to be significantly downregulated in skeletal and cardiac muscle of DM1 mice [92] and variably dysregulated in the skeletal muscle of older mdx mice and DMD patients [197]. Finally, miR-711 serves a protective function by promoting induction of anti-inflammatory pathways [172]. Specifically, miR-711 indirectly suppresses NLRP3 expression and transcriptional priming by inhibiting the NF-κB and FADD pathways [172]. This process is initially induced by adiponectin, a hormone that has emerged recently as a master regulator of inflammation in several tissues [172]. Interestingly, adiponectin seems to be overexpressed in mdx mice [198] but significantly decreased in DM1 patients [199], pointing to a potential rescuing role in some subtypes while being diminished in other subtypes (Fig. 2).

TNF is another classic pro-inflammatory agent yet its involvement in MD has been researched more recently. Typically associated with inflammation amplification, in the context of MDs the TNFs are more involved in the later stages of the inflammatory process: myonecrosis [200, 201]. This process is mainly initiated by the NF-κB pathway, which activates TNF [202]. TNF in turn induces miR-146a and miR-223 expression which is significant for several reasons [202]. These miRNAs are known to be heavily involved in inflammatory processes of disease. Additionally, IGF-1-induced upregulation of miR-16 has been shown to stimulate the degradation of TNF-α and subsequently lead to decreased presence of pro-inflammatory cytokines [141]. Importantly, TNF and its corresponding miRNAs have been shown to be markedly upregulated in mdx mice [203] and in DMD patients [204].

### Fibrosis

Fibrosis a degenerative process in which muscle fibers are replaced with fibroblasts, adipocytes, and ECM components [205]. It is considered one of the main hallmarks of MD and is involved in almost all the MD subtypes [206]. Fibro-adipogenic progenitors (FAPs) are a subset of mesenchymal stem cells that are typically quiescent in normal muscle but become activated upon muscle injury or in dystrophic conditions [207]. FAP activity is typically transient as it mainly functions to fibrose injured tissue to prevent any further damage, however in dystrophic conditions the FAP signal is maintained past the recovery threshold and instead becomes yet another irreversible pathological mechanism [207]. Fibrotic degeneration is typically a late-stage mechanism and therefore is most often observed and studied in end-stage patients and mice models [189]. This suggests the presence of preceding forces that influence, or even initiate, the fibrotic process. Indeed, there is a plethora of evidence suggesting that fibrosis occurs as a result of various earlier pathological mechanisms including unrelenting inflammation (e.g., macrophages, cytokines), impaired regenerative signaling (e.g., TGF-β pathway, renin-angiotensin system), and faulty ECM remodeling (e.g., via matrix proteases) [208]. In recent years, a subset of miRNAs called fibromiRs (e.g., miR-21, miR-29, miR-199a-5p, miR-214-3p) have gained notice in the field of muscle fibrosis [209] (Table 3).

**Fig. 3:**
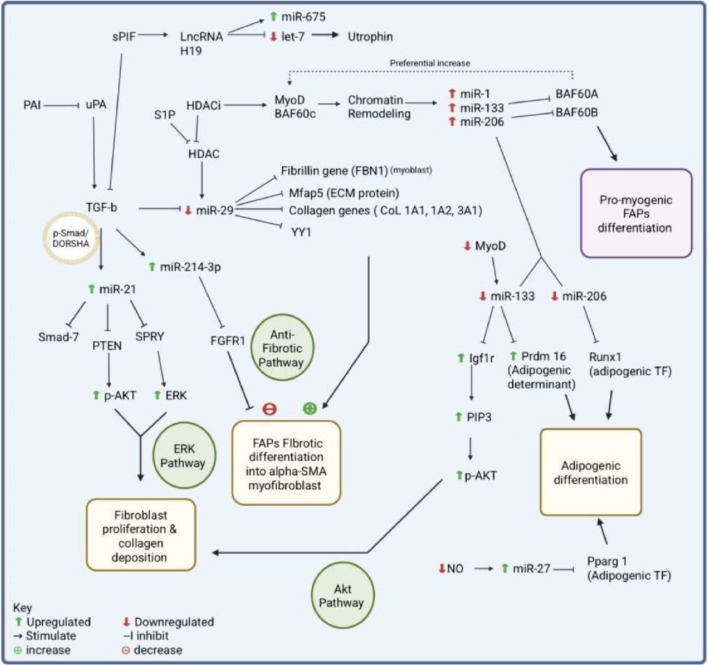
A network of interactions between ncRNAs and their direct targets guiding a complex interplay between fibrogenesis and adipogenesis. Abbreviations: *sPIF* synthetic preimplantation factor, *lncRNA* long non-coding RNA, *PAI* plasminogen activator inhibitor, *uPA* urokinase-type plasminogen activator, *S1P* sphingosine-1-phosphate, *HDACi* histone deacetylase inhibitor, *MyoD* myoblast determination protein 1, *BAF60A,* *BAF60B*, and *BAF60C* subunits of Brg1/Brm-associated factor 60 chromatin remodeling complexes, *TGF-b* transforming growth factor beta, *HDAC* histone deacetylase, *p-Smad* phosphorylated smad transcription factor, *YY1 *Yin yang1 transcriptional repressor, *PTEN* phosphatase and tensin homolog, *SPRY* sprouty gene, *p-AKT* phosphorylated AKT, *ERK *extracellular-signal-regulated kinase, *FAPs* fibro-adipogenic progenitor cells, *alpha-SMA* alpha smooth muscle actin, *Igf1r* insulin-like growth factor 1 receptor, *PIP3* phosphatidylinositol 3,4,5-triphosphate, *NO *nitric oxide, *TF* transcription factor

TGF-β is considered one of the main regulators of fibrosis due to its ubiquitous involvement in various stages and pathways. TGF-β-induced fibrosis is initiated by promoting the entry of SMADs into the nucleus where they serve as transcription factors of pro-fibrotic genes [210]. In young mdx muscle, fibroblasts overexpress PAI-1 (regulator of uPA and plasmin) which in turn prevents conversion of pro-TGF-β into its active form, thereby safeguarding the muscle from a perpetual cycle of TGF-induced fibrosis [164]. However, as the disease progresses, PAI-1 eventually loses its uPA-regulating capacity, which leads to uPA’s unhindered activation of TGF-β and the subsequent pro-fibrotic effects via SMAD gene regulation [164]. This process is mediated by miR-21 inhibition of PTEN and the subsequent overactivation of the PI3K/AKT pathway [164] (a well-established non-SMAD/TGF-β pathway involved in fibrosis [165]). The influence of miR-21 is demonstrated in studies where miR-21 downregulation in DMD muscles directly inhibits TGF-β1-induced transdifferentiation of fibroblasts to myofibroblasts while miR-21 upregulation promotes transdifferentiation [78] (Fig. 3). miR-21 has been shown to be elevated in various MD subtypes, including DMD, LGMD, FSHD, and DM [211], and therefore is a key culprit in this pathomechanism across the dystrophy spectrum.

On the other hand, miR-29 expression has been shown to inhibit various ECM component genes including collagen (e.g., COL1A1, COL1A2, COL3A1) and microfibrillar-associated proteins (Mfap-5), therefore serving as the countermeasure to miR-21’s fibrotic tendencies [79]. Decreased or generally dysregulated miR-29 expression would therefore entail potential fibrotic consequences due to loss of an important fail-safe mechanism. Indeed, studies have demonstrated significant downregulation in DMD, mdx, DM, and CMD muscle [78, 211]. Another TGF-β-induced miRNA, miR-214-3p, has been shown to modulate the FGF2 anti-fibrotic signaling pathway via downregulation of FGFR1 receptors to mediate α-SMA fibrotic activity [169]. This miRNA was shown to be upregulated in DMD and so is likely to contribute to its fibrotic phenomena [169]. In contrast, one study showed that miR-378-KO mdx mice exhibited diminished fibrosis, possibly as a result of the decreased FGF1 levels which was shown to be a target of miR-378 [46]. A previously mentioned study by Morgulis et al. showed that injection of sPIF targeted miR-675 and led to inhibition of TGF-β expression with subsequent decrease of tissue fibrosis in mdx mice [83].

The myomiRs have not traditionally been associated with muscle fibrosis; however, growing evidence suggests some degree of involvement. For example, investigators injected injured rats with myomiRs (miR-1, miR-133, miR-206) and discovered that this essentially prevented fibrosis via downregulation of TGF-β [72]. Subsequent studies have further elaborated on this finding by showing that miR-133 directly downregulates TGF-β by specifically targeting various myofibroblast differentiators (e.g., ɑ-SMA, CTGF, collagen) [212]. Another paper further developed this line of evidence by suggesting a link between miR-133 and one of TGF-β’s main effectors of fibrosis, myostatin [213]. Additionally, others demonstrated that deletion of miR-133 in mdx mice led to increased fibrosis which they determined was a result of the unhindered expression of TGF-β fibrosis effectors TGIF1, SMAD3, and SMAD5 [214]. Other investigators have expounded on the role of myomiRs in fibrosis by identifying a HDAC-myomiR-BAF60 network [161]. They showed that injecting young mdx mice with HDAC inhibitors (HDACi) induced the expression of the myomiRs which directly downregulate two BAF60 variants (BAF60A and BAF60B) [161]. This is important as they showed that these BAF60 variants directly regulated the fate of FAPs in dystrophic muscle by determining whether they adopt the myogenic lineage or the fibro-adipogenic lineage [161]. However, they have yet to elaborate on how, or if, this contributes in a significant way to fibrosis (or fatty degeneration) in general.

### Fatty infiltration/deposition

Fatty infiltration is the accumulation of fatty deposits inside muscle tissue, typically as a result of dysregulated FAPs [215]. This finding is present in several subtypes of MD including DMD [216], DM [217], CMD [218], LGMD [219], FSHD [220], and OPMD [221]. It is important to note that fatty infiltration and fibrosis are inextricably linked pathological phenomena in muscle due to the fact that they share common mesenchymal precursor cells [222], and therefore the ncRNAs that influence fibrosis will probably also be relevant to fatty infiltration (Fig. 3). Indeed, miR-206 is also involved in fatty infiltration as it targets Runx1, limiting its translation in FAPs [168]. miR-206 mimicking in mice reduces fatty infiltration and therefore the dysregulation of miR-206 in MD could very well contribute to the observed fatty degeneration [168]. Loss of MyoD—a common MD pathophysiology trend—leads to conversion of myoblasts to brown adipocytes through the upregulation of Prdm16, which is a key target of miR-133 [223]. Furthermore, MyoD^KO^ cells injected into mdx mice led to increased adipogenic markers [223].

### Autophagy

Autophagy, the process of cellular recycling via targeted cell breakdown, is one of the main ways by which the body regulates cell turnover. So far, studies have shown abnormal regulation of autophagy in almost all MDs [224–226]; however, very little has been revealed regarding the ncRNAs that contribute to this process. For example, miR-7 regulates autophagy with low levels of this miRNA promoting it and high levels repressing it [227]. Researchers have discovered that restoring miR-7 via agomiR-7 was sufficient in rescuing DM1 myoblast fusion defects and myotube growth [227]. One of the main proteins involved in this process is MBNL1, which directly regulates apoptosis via the mTOR pathway [228]. It is hypothesized that MBNL1 and miR-7 function in parallel, evidenced by the fact that upon silencing of a mutually associated protein, MSI2, a significant upregulation of both miR-7 and MBNL1 occurs with downregulation of atrophy-related genes and repression of excessive autophagy [229]. Moreover, administration of miR-23b and miR-218 antagonists in DM1-like phenotype Drosophila raised MBNL1 protein levels and improved muscular integrity, thereby suggesting a direct relationship between MBNL1 and miR-23b and miR-218 in the context of cell turnover homeostasis [230].

### Apoptosis

Although not as well researched as other pathological mechanisms in MD, apoptosis has nevertheless been shown to be a contributory force in several MDs including DM1, FSHD, CMD, OPMD, and Bethlehem Myopathy [231]. So far, it has mostly been reported as an aggressive “burst” event occurring in the early stages of dystrophy [231]. One important ncRNA classically involved in apoptosis of several tissues is miR-133, which directs apoptotic pathways via several proteins (e.g., caspase-3, LASP1) [167, 232]. In DM1, altered subcellular localization of miR-133b occurs which is thought to contribute to the apoptotic mechanisms in this subtype [233]. In regard to DMD, miR-486 inhibition led to increased caspase-3/7 levels which in turn resulted in increased cellular apoptosis [51]. This could be a viable pathomechanism as miR-486 is significantly downregulated in DMD patients [51]. DUX4 is also involved in apoptosis as it encodes a transcription factor that is toxic to many cell types, thereby leading to forced apoptosis, and miR-675 is a key regulator of DUX4 expression. When researchers induced DUX4 inhibition using miR-675, they discovered that this protected cells from apoptosis [171]. Additionally, they found that β-estradiol (previously linked to FSHD pathogenesis) is capable of simultaneously increasing miR-675 and reducing DUX4 expression [171]. Another important relationship is that of FAIM2 and miR-3202, where researchers have shown that inhibition of miR-3202 leads to upregulation of FAIM2 and subsequently less apoptosis [173]. Interestingly, an inverse relationship exists between DUX4 and FAIM2 in which increased expression of one leads to reduced expression of the other [173]. As such, the investigators suggest that miR-3202 inhibition is protective against DUX4’s apoptotic forces through upregulation of FAIM2 [173].

### Calcium influx

Intracellular calcium is vital to various muscle tissue functions including muscular contraction [234], myoblast differentiation [235], and energy homeostasis [236]. Interestingly, it has been shown in mice experiments that Ca^2+^ dysregulation—independent of membrane fragility—is sufficient to cause a dystrophic phenotype [237]. Indeed, Ca^2+^ dysregulation has been directly implicated in DMD [238], LGMD [239], and DM [240]. miR-1 hyperactivates RyR2 (a prominent Ca^2+^release channel) by inhibiting the PP2A regulatory subunit B56α which leads to excessive Ca^2+^ leak [163]. This is a highly relevant pathomechanism in DMD as treatment of mdx mice with RyR stabilizers attenuates the disease phenotype [241] and treatment of DMD cell culture myoblasts with RyR stabilizers enhanced efficacy of exon-skipping drugs [242]. miR-25 may also be relevant due to the fact that it targets sarcoendoplasmic reticulum calcium ATPase (SERCA2a) mRNA [166]. Overexpression of the SERCA pump has been shown to significantly ameliorate DMD-associated DCM in mdx mice [243] and miR-25 inhibition in heart failure mouse models has led to improved cardiac outcomes [244]. However, the direct effects of miR-1 and miR-25 in the context of Ca^2+^ channel dysregulation in dystrophic muscle has yet to be reported. On the other hand, a study of Ca^2+^ pathways in calpainopathies found that miR-184 is significantly downregulated in LGMD2B patients and suggested that this downregulation could reflect the presence of inflammatory processes that exacerbate LGMD2B dystrophic muscle [158].

### Oxidative stress

Reactive oxygen species (ROS) are free radicals that naturally form in various tissues and normally contribute to several physiological functions [245]. However, an excessive accumulation of ROS due to perturbation of antioxidant enzymes such as superoxide dismutase, catalase, and peroxidase, leads to devastating long-term oxidative damage [245]. Oxidative stress is widely regarded as a key effector of several MDs [246] including DMD [247], FSHD [248], LGMD [249], DM [250], EDMD [251], CMD [252], and OPMD [253]. One study has attempted to link miR-1 with the ROS-regulator, glucose-6-phosphate dehydrogenase (G6PD), and the epigenetic regulator, HDAC2, in the context of the rampant oxidative stress observed in mdx mice [162]. They showed that the decreased miR-1 levels in mdx mice correlated with increased G6PD expression as well as a decrease in antioxidant glutathione levels [162, 254]. Increased G6PD would normally increase its downstream effector, NADPH, which would lead to increased glutathione and ultimately enhanced protection against free radical damage. However, in mdx mice, they found that most of the NADPH was utilized in the activation of NADPH oxidase (NOX) which led to conversion of superoxide species into peroxinitrite [162]—a highly reactive oxidant that causes direct DNA damage [255]. Furthermore, they found that increased peroxinitrite caused deficient nitrosylation of HDAC2, which prevented it from performing its regular DNA protecting functions [162]. As such, they demonstrated that dysregulated miR-1 levels mediate perpetual oxidative stress damage in mdx mice. Another study links the myomiRs (miR-1-3p, miR-133a-3p, and miR-206) and a set of oxidative-stress-related miRNAs (“OS-R”: miR-21-5p, miR-31-5p, miR-128-3p, and miR-144-3p) to the ROS-associated natural history of DMD, and indeed found that all of these miRNAs (except for miR-1-3p) were significantly lowered in non-ambulatory patients compared to patients who were still ambulatory [256]. Lastly, a study determined that miR-200c, previously shown to be upregulated in mdx mice, was associated with increased oxidative stress and subsequent dystrophic effects via a p66Shc-dependent mechanism in DMD [38].

## Signaling failure

Biochemical signaling within and between cells is the method by which communication of information is transferred in the body. Unsurprisingly, “signaling failure” plays a key role in various pathomechanisms in MD owing to its systemic and widely dispersed effects on all communication pathways both within and beyond the muscle [257]. Signaling pathway failure has been associated with structural instability, myogenesis perturbations, genetic dysregulation, improper cell membrane potential, defective repair processes, and more [257, 258]. There are various established signaling pathways contributing to MD pathophysiology, such as DGC pathway, Notch pathway, NF-κB pathway, JAK-STAT pathway, Wnt pathway, PTEN/Akt pathway, IGF-1 pathway, and several more. This section will attempt to summarize the main pathways, their ncRNA-protein clusters, and the various associated pathomechanisms in the context of MD (Table 4).

**Table 4:** A summary of the most prominent ncRNAs and their direct targets in signaling failure

ncRNA	Target	Type of MD/cell	**Pathological role**	**References**
miR-16	TNF-a	DMD	Reduces inflammation	[141]
miR-34c	nNOS	DMD	Nitric oxide signaling	[89]
	Notch1	PSCs	Regulates skeletal muscle development	[259]
miR-140	Notch1	Zebrafish	Suppresses myogenesis and maintains MuSC quiescence	[260]
miR-146a	Numb	C2C12	Delays myogenic differentiation	[32]
miR-222	B1-syntrophin	DMD	Regulation of DAPC	[261]
miR-449a	Jag1	C2C12 Human primary skeletal muscle cells	Inhibition of Notch signaling	[262]
miR-708	nNOS	DMD	Nitric oxide signaling	[89]

*miR* microRNA, *DMD* Duchenne muscular dystrophy, *PSCs* porcine muscle satellite cells, *IL*-*34* interleukin 34, *SC* satellite cells, *KO* knockout, *nNOS* neuronal nitric oxide synthase, *MD* muscular dystrophy, *DAPC* Dystrophin associated protein complex, *MuSC* muscle satellite cell

### DGC signaling

The DGC is a set of sarcolemmal proteins that serve both a structural and a signaling role in the muscle niche [263]. It is primarily composed of the following proteins: dystrophin, DGs, dystrobrevins, sarcoglycans, syntrophins, sarcospan, caveolin-3, and nitric oxide synthase (NOS) [263].

**Fig. 4:**
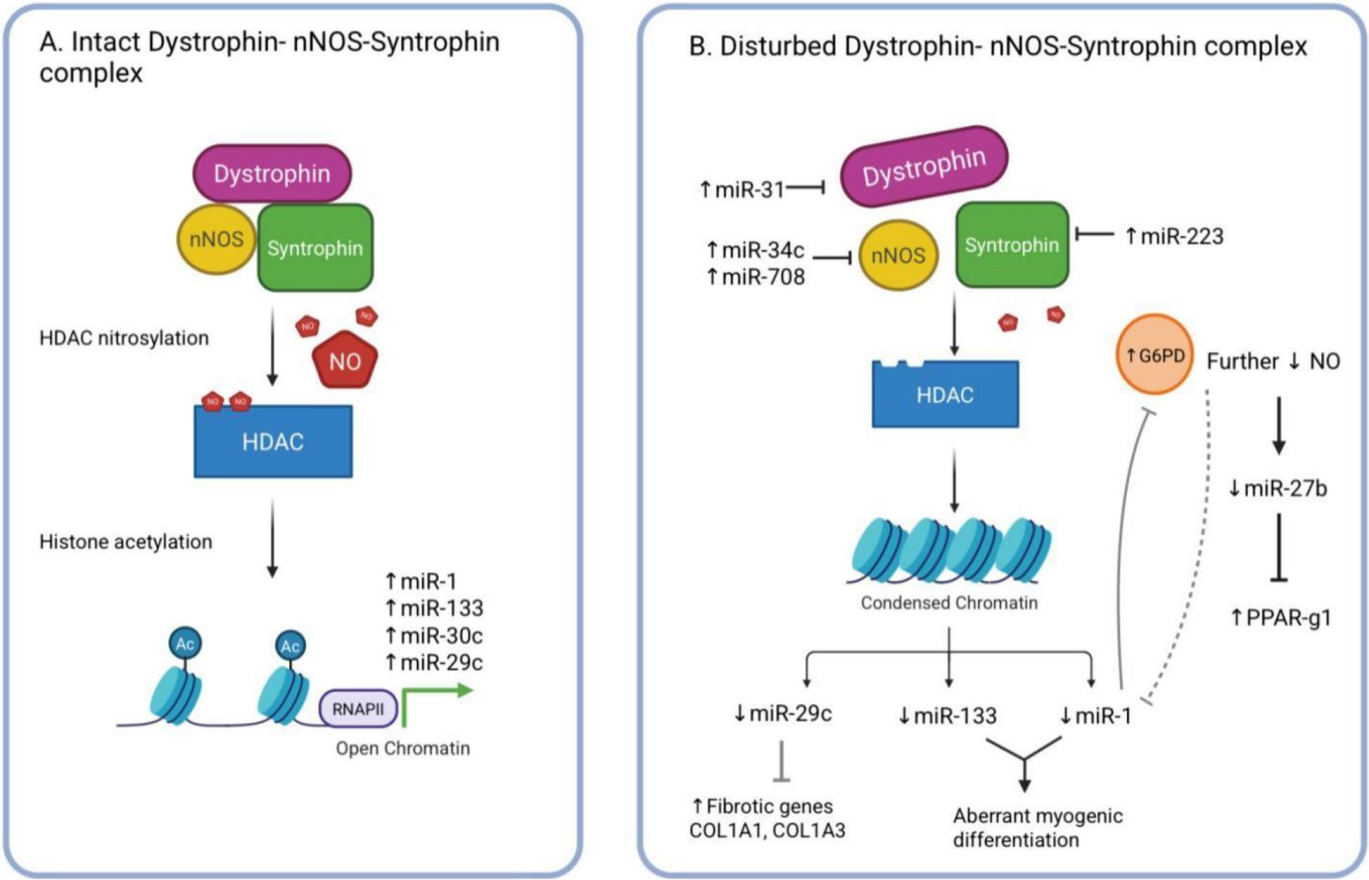
Dystrophin-nNOS-Syntrophin complex. **a** Integral interaction among dystrophin, nNOS, and syntrophin, a member of the dystrophin-associated protein complex, leads to up-regulation of miR-1, miR-133, miR-30c, and miR29c via HDAC-dependent mechanism. **b** Several miRNAs regulate dystrophin-nNOS signaling which in turn regulates implicated miRNAs such as miR-133/miR-1, and miR-29c involved in various pathological circuits in DMD including muscle regeneration, and fibrogenesis, respectively. Abbreviations: *miR* microRNA, *nNOS* neuronal nitric oxide synthase, *NO* nitric oxide, *HDAC *histone deacetylase, *Ac* acetylation, *RNAPII* RNA polymerase II, *PPAR-g1* peroxisome proliferator-activated receptor gamma 1, *G6PD *glucose-6-phosphate dehydrogenase, *COL1A1* and *COL1A3* collagen type I alpha 1 and 3 chain

The DGC-nNOS signaling pathway is a vital process involved in several muscle homeostatic measures [264]. In healthy muscle, the DGC recruits nNOS to the sarcolemma which leads to production of nitric oxide (NO) which is relevant to MD for its roles as both a free radical and a regulator of multiple downstream targets [265]. Multiple DGC components are involved in this process: syntrophin localizes nNOS to the sarcolemma [266], caveolin-3 regulates nNOS function by competing with calmodulin [267] (a nNOS activator [268]), and dystrophin maintains nNOS function by regulating the free radical status through various means [162] (Fig. 4).

With regards to syntrophin, it has been shown that inducing oxidative stress in C2 myoblasts leads to upregulation of α-syntrophin and that α-syntrophin-overexpressing cells are resistant to oxidative stress [269]. The aberrant splicing of α-dystrobrevin in DM1 muscle has been shown to significantly elevate α-syntrophin expression [270]. However, whether α-syntrophin increases *due* to oxidative stress or as a means of protecting *against* oxidative stress has yet to be determined. miR-222, which is normally downregulated in myogenic cells [40], targets β1-syntrophin and is significantly elevated in dystrophic muscle [261]. Furthermore, anti-miR-222 treatment led to increased β1-syntrophin rescue [261]. The authors of this study suggest that β1-syntrophin’s signaling capacity is disturbed in DMD and that β1-syntrophin targeting could potentially aid in dystrophy treatment [261].

On the other hand, the dystrophin absence in DMD inhibits the DGC-nNOS pathway which entails a downstream pathological cascade: dystrophin deficiency downregulates and delocalizes nNOS, which alters the nitrosylation of HDAC2, causing defective release of HDAC2 from its chromatin hold, and ultimately preventing NO production [162]. Simultaneously, the impaired HDAC2 nitrosylation causes ineffective release of HDAC2 from certain miRNA promoters (miR-1, miR-133, and miR-29c). When these miRs are not properly recruited, oxidative stress goes unchecked (as the absence of miR-1 disrupts the feedforward cycle with the ROS-regulating G6PD molecule) and fibrosis increases (via upregulation of pro-fibrotic genes such as COL1A1 and COL1A3) [162]. The importance of this pathway is further evidenced by the fact that exon-skipping treatment of mdx mice led to dystrophin restoration, proper nNOS–dystrophin associated protein complex (DAPC) colocalization, and general amelioration of the dystrophic phenotype [162]. Additionally, miR-708-5p and miR-34c-5p have been shown to modulate nNOS and are overexpressed in BMDd_45–55_ muscle biopsies, in DMDd_45–52_ myoblasts, and in mdx mice, however their relation to the DGC has not been elucidated [89].

The dystroglycan-dystrophin-syntrophin1 (Dg-Dys-Syn1) axis of the larger DGC signaling complex is of particular interest as it regulates expression of various miRNAs [271] and perturbations of its individual component levels seem to be highly interrelated [272]. One study found that in Drosophila MD models, even though the following miRs are directly regulated by separate proteins in the Dys-Dg-Syn1 axis, appropriate levels of miR-252, miR-956, and miR-980 depend on the collective integrity of the whole axis (i.e., perturbation of other indirectly-associated proteins could cause perturbations to non-direct miRNA targets) [271]. This means that perturbation of any of the Dys-Dg-Syn1 axis results in the dysregulation of these miRNAs. Intriguingly, miR-252 and miR-980 are themselves regulators of Dys, Dg, and Syn1 proteins too, which suggests a potential highly interconnected feedback loop that can easily go awry given the known scaffolding protein mutations that occur in MDs [271]. To further add to this, miR-252 is required for proper muscle development, and so perturbations of Dys-Dg-Syn1-miRNA networks will, at the very least, lead to improper myogenesis [271].

### Notch signaling

In the context of muscles, Notch signaling is mainly involved in maintaining a viable quiescent progenitor cell pool through various anti-myogenic activities [273]. As such, any perturbation of this signaling system is bound to have downstream effects on muscle cell content. Indeed, mutations of several Notch-affiliated proteins (including Jag1-2, MEGF10, and POGLUT1) have been reported as key contributors to various MD subtypes [274].

miR-140 has been shown to target multiple members of the Notch signaling pathway (e.g., DNER, JAG1, HEY1, NUMB) [260] and is also significantly deregulated in DM1 patients [275]. miR-146 is another ncRNA that targets Numb which promotes satellite cell differentiation by inhibiting Notch signaling. miR-146 inhibition rescued Numb expression and led to differentiation. This miRNA is significantly increased in mdx mice [211] as well as FSHD and LGMD patient sera [59, 105]. Furthermore, miR-449a targets Jag1 and ultimately inhibits Notch signaling which is evidenced by the decreased levels of Notch downstream targets (e.g., Hes1 and Hey1) [262]. miR-449a-mediated Notch inhibition was investigated in several cohorts (e.g., mdx mice, DMD patients, newborn mice) where mdx mice responded best to this intervention with overall improved phenotype [197]. The differentiation inducer, Mef2c, has been shown to induce the myomiRs miR-1 and miR-206 in the process of downregulating Notch3 (which itself decreases differentiation) [276]. However, the MEF2 protein family (including MEF2C) is severely altered in muscle biopsies of DM patients, possibly suggesting impaired function as a differentiation regulator via Notch3 suppression [277]. miR-206 also negatively regulates Notch3 and so the absence of miR-206 has been shown to increase Notch3 levels and delay muscle regeneration in CTX-injured mdx mice [66]. miR-34c is a component of a self-perpetuating regulatory loop involving Notch1 [259]. One in vivo animal model study showed that Notch1 directly reduced the transcription of miR-34c but also described Notch1 as being a direct target gene of miR-34c [259]. A potentially vicious cycle of impaired Notch signaling may occur in the dystrophic context as is evidenced by the overexpression of miR-34c and its subsequent dysregulation of nNOS in DMD myoblasts and BMD biopsies [89].

### NF-κB pathway

NF-κB is an essential group of transcription factors that are involved in many cellular activities including cell growth, apoptosis, and inflammatory responses. In disease conditions, NF-κB contributes significantly to muscle degradation and atrophy in various ways, e.g., the augmentation of ubiquitin–proteasome system proteins (namely E3) which results in the degradation of specific muscle proteins; increased expression of molecules associated with inflammatory mechanisms that directly or indirectly result in muscle wasting; and disruption of the myogenic differentiation program thereby altering the regeneration mechanisms of newly-atrophied muscles [278].

During homeostasis, NF-κB remains inactive due to binding of reversible protein inhibitors (IκBs) [279]. TNF-α expression activates NF-κB by phosphorylating IκB-α, thereby neutralizing its inhibitory powers over NF-κB [279]. This is normally a transient process, but in dystrophic muscle TNF-α presence is sustained, thereby perpetuating a vicious cycle of NF-κB activation and cellular destruction [280, 281]. miR-223 has been shown to downregulate both TNF-α and NF-κB, thereby diminishing the destructive effects of perpetual NF-κB activation [196, 282]. However, miR-223 was shown to be significantly downregulated in DM1 mice compared to controls, possibly suggesting that this cycle goes unchecked in DM [92]. In vivo inhibition of IκB-α and subsequent silencing of NF-κB ameliorated mdx disease phenotype [283], and there are various miRNAs associated with this protein in other tissues [284], but none yet reported in muscle.

Several NF-κB proteins are involved in muscle wasting including MuRF1 and atrogin-1 via the ubiquitin–proteasome (UPS) pathway. Indeed, perturbations of the UPS pathway, and its associated proteins MuRF1 and atrogin-1 have been shown to occur in several MDs including dysferlinopathies and DM [179, 285]. One of the key influencers of this process is miR-7. miR-7 was shown to be significantly downregulated in a Drosophila model of DM1—which exhibited characteristic signs of UPS-induced wasting—and miR-7 restoration led to amelioration of the disease phenotype [227].

The NF-κB–TNF axis could also be involved in the perturbation of myogenesis in MD. For example, in a series of experiments, one study found that the TNF signaling was the only pathway that was simultaneously significantly upregulated in DM1 mouse and inhibited after overexpression of miR-322/−503 [286]. Moreover, they found that inhibition of TNF by this miRNA cluster led to enhanced myogenesis in DM1 mice [286]. They also suggested that the persistent inflammatory state of DM1 (in essence due to the perpetual NF-κB–TNF axis) was one of the major causes of this impaired myogenesis—thereby tying the two separate pathomechanisms together [286].

One study identified of group of miRNAs that are linked to the NF-κB signaling pathway [287] (Fig. 2). The proposed “inflammatory miRNAome” consists of miR-142-3p, miR-142-5p, miR-146a, miR-301a, miR-324-3p, miR-455-3p, miR-455-5p, miR-497, and miR-652 [287]. The researchers suggested that all these miRNAs are elevated in mdx mice (except for miR-324-2p which showed no difference compared to wildtype) [287]; however, other papers seem to suggest a contradicting stance. In separate studies, only miR-142-5p and miR-146a were shown to be upregulated in mdx and GRMD animal model muscle [33, 288]. On the other hand, only miR-455-5p was increased in FSHD and LGMD2A biopsies [59], miR-652 levels were shown to be no different in DM1 muscle than that of healthy controls [289], and the rest of the miRNAs were not mentioned at all in the literature pertaining to MD. Furthermore, the paper by Fiorillo et al. shows that the DNA promoters of eight out of the nine miRNAs contain one or more DNA sites that are bound by NF-κB [287]. Additionally, the paper provides evidence that two NF-κB-inhibiting drugs (prednisolone and vamorolone) were capable of returning all these miRNAs (with the exception of miR-301a) to wild-type levels in mdx mice [287]. Another study also showed that prednisolone/vamorolone treatment was capable of restoring DTMs (including the previously mentioned miR-146a and the novel miR-233) back to wildtype levels in vivo [33].

## Conclusion

MD is a large set of conditions characterized by progressive muscle weakening and wasting that occurs secondary to genetic and epigenetic influencers, of which the ncRNA factor seems to be gaining traction as one of the main culprits of this incurable disease. It is an extremely complex and multifactorial pathology involving various hard-to-treat issues such as impaired regeneration, self-sabotaging pathomechanisms, inefficient safeguards, perturbed communications, and many more. From the literature, we extrapolated four main themes that seem to be prevalent across all MD subtypes: myogenesis insufficiency, structural instability, destructive tendencies, and signaling failure. We then attempted to describe some of the key ncRNA clusters (e.g., myomiRs, dystromiRs, fibromiRs, DTMs) as well as some notable individual standouts (e.g., miR-17, miR-711, lncRNA H19)—with their associated protein effectors—that are involved in the various unique pathophysiological profiles of different MD subtypes. This article sheds light on the innately complex nature of MD and provides key insights into the pathophysiological mechanisms which can hopefully one day be exploited and manipulated for therapeutic purposes.

## Data Availability

Not applicable.

## Abbreviations

AC: Acetylation
alpha-SMA: Alpha smooth muscle actin
α-DG: Alpha dystroglycan
Α-Syn: Alpha-syntrophins
β-DG: Beta-Dystroglycan
β-Syn: Beta-Syntrophins
BMD: Becker muscular dystrophy
Ca2^+^: Calcium
CirRNA: Circular RNA
CMD: Congenital MD
CPM: Chicken primary myoblast
CRD: Cysteine-rich domain
CTD: Carboxy-terminal domain
DAPC: Dystrophin-associated protein complex
DCM: Dilated cardiomyopathy
DG: Dystroglycan
Dg-Dys-Syn1: Dystroglycan-dystrophin-syntrophin 1
DGC: Dystrophin glycoprotein complex
DM: Myotonic dystrophy
DM1: Myotonic muscular dystrophy
DMD: Duchenne muscular dystrophy
DMD-iCMs: DMD patient-induced pluripotent stem cell-derived cardiomyocytes
DTMs: Dystrophin targeting miRNAs
ECM: Extracellular matrix
EOMs: Extraocular muscles
ERK: Extracellular-signal-regulated kinase signaling
FADD: FAS-associated death domain protein
FAPs: Fibro-adipogenic progenitors
FCMD: Fukuyama congenital muscular dystrophy
FSHD: Facioscapulohumeral muscular dystrophy
G6PD: Glucose-6-phosphate dehydrogenase
GR: Glucocorticoid receptor
GRMD: Golden retriever muscular dystrophy
HDAC: Histone deacetylase
HDACi: Histone deacetylase inhibitor
Igf1r: Insulin-like growth factor 1 receptor
IL-18: Interleukin-18
IL-1β: Interleukin-1 beta
IL-34: Interleukin 34
Jak: Janus kinase
KO: Knock out
LAMA2: Laminin α2-chain gene
LGMD2A: Limb–girdle muscular dystrophy 2A
LGMD2B: Limb–girdle muscular dystrophy 2B
Lnc: Long non-coding RNA
LncRNA: Long non-coding RNA
MD: Muscle dystrophies
MDC1A: Merosin-deficient congenital muscular dystrophy
MEB: Muscle-eye-brain diseases
MiR: MicroRNA
MiRNA: MicroRNA
MM: Mitochondrial myopathy
mRNP: Messenger ribonucleoprotein
MuSC: Muscle satellite cell
myomiRs: Muscle-enriched miRNAs
MyoR: Musculin
NcRNA: Non-coding RNA
NFkB: Nuclear factor kappa
NLRP3: Inflammasome
nNOS: Neuronal nitric oxide synthase
NO: Nitric oxide
NOS: Nitric oxide synthase
NOX: NADPH Oxidase
NTD: Amino-terminal domain
OPMD: Oculopharengeal muscular dystrophy
p-AKT: Phosphorylated AKT
P-Smad: Phosphorylated smad transcription factor
P21: CDKN1
P27: CDKN1B
PAI: Plasminogen activator inhibitor-1
PIP3: Phosphatidylinositol 3,4,5-triphosphate
PPAR-g1: Adipogenic transcription determinant
PSCs: Porcine muscle satellite cells
PTEN: Phosphatase and tensin homolog
RNAPII: RNA polymerase I
RyR2: Ryanodine receptor
S1P: Sphingosine-1-phosphate
SC: Satellite cell
SiRNA: Small interfering RNA
sPIF: Synthetic preimplantation factor
SPL: Sphingosine phosphate lyase
SPRY: Sprouty gene
SRF: Serum response factor
STAT: Signal transducers and activators of transcription
STAT6: Signal transducer and activator of transcription family of proteins
TGF-b: Transforming growth factor beta
uPA: Urokinase-type plasminogen activator
WWS: Walker–Warburg syndrome
YY1: Yin yang1 transcriptional repressor
